# Inhibiting O-GlcNAcylation impacts p38 and Erk1/2 signaling and perturbs cardiomyocyte hypertrophy

**DOI:** 10.1016/j.jbc.2023.102907

**Published:** 2023-01-13

**Authors:** Kyriakos N. Papanicolaou, Jessica Jung, Deepthi Ashok, Wenxi Zhang, Amir Modaressanavi, Eddie Avila, D. Brian Foster, Natasha E. Zachara, Brian O'Rourke

**Affiliations:** 1 Division of Cardiology, Department of Medicine, The Johns Hopkins University School of Medicine, Baltimore, Maryland, USA; 2 Department of Biological Chemistry, The Johns Hopkins University School of Medicine, Baltimore, Maryland, USA; 3 Department of Oncology, The Johns Hopkins University School of Medicine, Baltimore, Maryland, USA

**Keywords:** OGT, OGA, Tab1, Hsp90, Hsp27, Creb, Gata4, MKK3/6, OSMI, TMG, ConA, Concanavalin A, MAPK, mitogen-activated protein kinase, NOX, NADPH oxidase, NRVM, neonatal rat ventricular myocyte, O-GlcNAc, O-linked GlcNAc, OGA, O-GlcNAcase, OGT, O-GlcNAc transferase, PE, phenylephrine, ROS, reactive oxygen species, TMG, Thiamet G

## Abstract

The dynamic cycling of O-linked GlcNAc (O-GlcNAc) on and off Ser/Thr residues of intracellular proteins, termed O-GlcNAcylation, is mediated by the conserved enzymes O-GlcNAc transferase (OGT) and O-GlcNAcase. O-GlcNAc cycling is important in homeostatic and stress responses, and its perturbation sensitizes the heart to ischemic and other injuries. Despite considerable progress, many molecular pathways impacted by O-GlcNAcylation in the heart remain unclear. The mitogen-activated protein kinase (MAPK) pathway is a central signaling cascade that coordinates developmental, physiological, and pathological responses in the heart. The developmental or adaptive arm of MAPK signaling is primarily mediated by Erk kinases, while the pathophysiologic arm is mediated by p38 and Jnk kinases. Here, we examine whether O-GlcNAcylation affects MAPK signaling in cardiac myocytes, focusing on Erk1/2 and p38 in basal and hypertrophic conditions induced by phenylephrine. Using metabolic labeling of glycans coupled with alkyne-azide “click” chemistry, we found that Erk1/2 and p38 are O-GlcNAcylated. Supporting the regulation of p38 by O-GlcNAcylation, the OGT inhibitor, OSMI-1, triggers the phosphorylation of p38, an event that involves the NOX2–Ask1–MKK3/6 signaling axis and also the noncanonical activator Tab1. Additionally, OGT inhibition blocks the phenylephrine-induced phosphorylation of Erk1/2. Consistent with perturbed MAPK signaling, OSMI-1–treated cardiomyocytes have a blunted hypertrophic response to phenylephrine, decreased expression of cTnT (key component of the contractile apparatus), and increased expression of maladaptive natriuretic factors Anp and Bnp. Collectively, these studies highlight new roles for O-GlcNAcylation in maintaining a balanced activity of Erk1/2 and p38 MAPKs during hypertrophic growth responses in cardiomyocytes.

The enzyme O-GlcNAc transferase (OGT) utilizes UDP-GlcNAc as a substrate and covalently links the GlcNAc moiety to Ser/Thr residues of intracellular proteins (1, 2, 3). The O-GlcNAcase (OGA) catalyzes the opposing reaction, removing O-GlcNAc from proteins (4, 5). Collectively known as O-GlcNAcylation, this protein modification is reversible, highly dynamic, and positioned at the intersection of homeostatic and stress pathways (6, 7). Both *Ogt* and *Oga* genes, necessary for O-GlcNAc cycling, are essential for mammalian development and survival (8, 9, 10). In the heart and the cardiac myocyte, O-GlcNAc cycling has been shown to play important roles in ischemia-reperfusion injury (11, 12), hypertrophic dysfunction (13, 14, 15, 16), diabetic arrhythmias and cardiomyopathy (17, 18, 19), and heart failure (20, 21). The current view suggests that transient elevation of O-GlcNAcylation serves a protective role against stress, whereas prolonged increases exacerbate cardiac pathology (22, 23, 24, 25). Furthermore, it has been postulated that highly dynamic O-GlcNAcylation overlaps with phosphorylation to provide an additional layer of regulation of protein function (26). Focused studies in the heart and cardiac myocytes found that O-GlcNAcylation impacts the function of diverse kinases such as the Ca^2+^/calmodulin-dependent kinase CaMKII (27), the AMP-dependent protein kinase (28), and the growth regulator mTOR (29).

The mitogen-activated protein kinases (MAPKs) are a conserved family of kinases forming an ordered signaling cascade (MAP3K/MAP2K/MAPK), activated by mechanical and neuroendocrine factors acting on G protein–coupled receptors (30, 31). The main end-effectors of MAPK signaling, Erk1/2, p38α-δ, and Jnk1-3, have unique and overlapping effects in stressed cardiac myocytes, leading to proliferation, growth, and cell death. Erk1/2 mediate cardiomyocyte proliferation and survival (32, 33, 34) and are also important in physiological hypertrophy and proper structural organization of growing myofibrils (35, 36, 37, 38). The Gαq agonist phenylephrine (PE) acts through the small G protein Ras to activate the MAP3K Raf, which phosphorylates and activates MAP2Ks MEK1/2, upstream activators of Erk1/2 (39). Downstream targets of Erk1/2 are the ribosomal S6 kinases Rsk1-4 and Msk1/2 (40), which are kinases that relay the signal downstream to the translation machinery but also to transcription factors, such as Gata4, Elk-1, c-Myc, and Creb (41, 42, 43, 44, 45).

While Erk1/2 are thought to be associated with adaptive physiological responses, p38 signaling kinases are associated with inflammation, maladaptive remodeling, and reduced cardiac contractility (46, 47, 48). Gαq agonists mildly promote the phosphorylation of p38 through parallel mechanisms that involve phospholipase C and proteins Gβγ, which in turn activate the Rho family of GTPases (RhoA, Rac, Cdc42), ultimately activating MKK3/6 and p38 (49, 50, 51). The downstream targets of p38 include kinases Msk1, Mk2/3, and Mnk1/2 that, in turn, phosphorylate their own downstream targets such as the small heat shock protein Hsp27, the translation initiation factor eIF4E, and the transcription factors Elk-1, C/EBPβ, and Creb (49, 52). Some evidence indicates a direct interplay between O-GlcNAcylation and phosphorylation on transcription factors c-Myc, Creb, C/EBPβ (53, 54, 55), and Hsp27 (56, 57, 58). However, direct evidence for O-GlcNAcylation on upstream MAPKs, *e.g.*, on Erk1/2 and p38, is sparse.

In the present work, we examined whether short-term changes in O-GlcNAcylation could affect basal and stimulated MAPK signaling in cardiac myocytes. Our data indicate that decreasing O-GlcNAcylation with the OGT inhibitor OSMI-1 induces basal p38 phosphorylation at the early phase, whereas it blocks Erk1/2 phosphorylation and causes growth impairment at a later phase. We found several MAPK signaling members that could be putative targets for O-GlcNAcylation, including Erk1/2 and p38, but also upstream (MEK1/2, Tab1) and downstream mediators (Hsp27 and Creb). Our data reveal the widespread and intricate relationship between O-GlcNAcylation and MAPK signaling in cardiac myocytes, paving the way for further investigations into the role of O-GlcNAcylation in MAPK-related pathophysiology, including cardiac hypertrophy and heart failure.

## Results

### Contribution of MAPKs Erk1/2 and p38 to the hypertrophic response induced by PE

**Figure 1:**
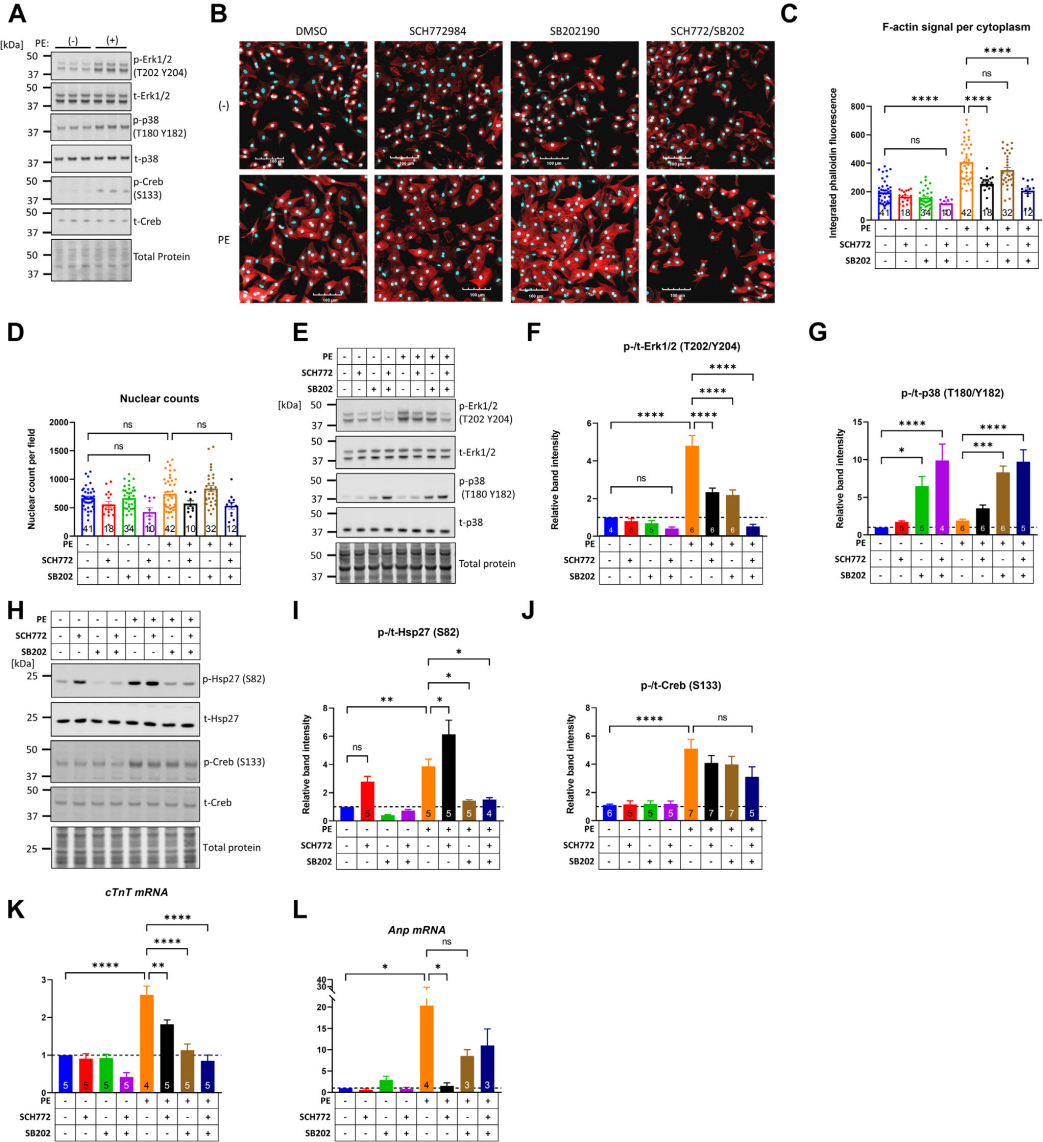
**Phenylephrine activates MAPKs Erk1/2 and p38 and their inhibition impairs cardiomyocyte growth.***A*, primary neonatal rat ventricular myocytes (NRVMs) incubated for 24 h in medium without growth factors were stimulated with 5 μM phenylephrine (PE) and harvested 30 min later for Western blot analysis. Phosphorylation of Erk1/2, p38, and Creb was assessed with their respective phospho-specific and total antibodies. *B*, NRVMs were treated with the Erk1/2 inhibitor SCH772984 (10 μM), the p38 inhibitor SB202190 (10 μM), or both and 6 h later, they were exposed to 5 μM PE. After 24 h of combined treatment, the cells were fixed and stained with phalloidin-Alexa 594 to detect and quantify fiber actin (F-actin) as index of NRVM cell size (hypertrophy). The scale bar represents 100 μm. *C*, per cytoplasm integrated signal intensity of phalloidin stain and (*D*) total number of nuclei identified per field of view. The data in (*C* and *D*) were extracted from confocal images imported into Cell Profiler. For the cytoplasmic F-actin signal, the mean value across a given field of view containing ∼850 cells is represented as a single data point. The number of fields quantified in each treatment group are shown in their respective bars. Bars represent means ± standard error. Comparisons across treatment groups were done with one-way ANOVA and Tukey post hoc test. ∗∗∗∗*p* < 0.0001; ns; not significantly different. *E*–*J*, representative Western blots and band intensity quantifications resulting from the indicated total and phospho-specific blots. Cells were pretreated for 6 h with 10 μM of the indicated inhibitors and followed by 30 min stimulation with 5 μM PE. The bar graphs represent mean band intensities averaged across the indicated number of biological replicates. *K* and *L*, cTnT and Anp mRNA expression across the indicated treatment groups. Cells were pretreated for 6 h with 10 μM of the indicated inhibitors followed by 24 h of stimulation with 5 μM PE before RNA isolation and cDNA synthesis for quantitative PCR. Numbers of biological replicates are shown in their respective bars. Bars represent means ± standard error. Comparisons across treatment groups were done with one-way ANOVA and Tukey post hoc test. ∗*p* < 0.05, ∗∗*p* < 0.01, ∗∗∗*p* < 0.001, ∗∗∗∗*p* < 0.0001, ns; not significantly different. MAPK, mitogen-activated protein kinase; NRVM, neonatal rat ventricular myocyte; PE, phenylephrine.

Neonatal rat ventricular myocytes (NRVMs) provide a cellular model to investigate the role of the MAPK signaling cascade in cardiac hypertrophy. Exposure to the alpha-1 adrenergic receptor agonist PE (5 μM, 30 min) increased the phosphorylation of Erk1/2, p38, and the downstream target Creb (Fig. 1*A*). To investigate the PE-induced hypertrophic signaling, we employed SCH772984 and SB202190, chemical inhibitors to kinases Erk1/2 and p38α/β, respectively (59, 60, 61). Hypertrophic growth was assessed at 24 h of PE stimulation by staining for actin using phalloidin (Fig. 1*B*). As shown in Figure 1, *B* and *C*, PE increased NRVM size (2.1-fold increase, *p* < 0.001), and this was reduced by the Erk1/2 inhibitor SCH772984 (50% reduction, *p* < 0.001). The p38 inhibitor SB202190 did not significantly affect PE-induced growth, while the combination of the two strongly suppressed PE-induced NRVM growth (Fig. 1*C*). The total numbers of nuclei per random field imaged were not significantly different across groups (Fig. 1*D*).

SCH772984 was potent in reducing the PE-induced phosphorylation of Erk1/2 and, interestingly, this response was also recapitulated by SB202190 (Fig. 1, *E* and *F*). In fact, the two inhibitors appeared to have an additive effect in suppressing PE-induced Erk1/2 phosphorylation (Fig. 1*F*). In cells treated with the p38 inhibitor SB202190, we observed a paradoxical increase in p38 phosphorylation. This, however, can be explained by considering that the phosphorylation of p38 is regulated by a feedback circuit, where inhibition of p38 derepresses its upstream kinase TAK1, leading to compensatory phosphorylation of p38, despite inhibition of its activity (62). Furthermore, the pyridinyl imidazole p38 inhibitors (*e.g.*, SB202190 or SB203580) block p38 at the active site of the kinase without impacting its phosphorylation (63). These two effects together explain the increased p38 phosphorylation with SB202190 in basal and PE-treated conditions (Fig. 1, *E* and *G*).

A downstream target of p38, MK2, phosphorylates the small heat shock protein Hsp27. We confirmed the functional inhibition of p38 by examining the phosphorylation of Hsp27. As expected, PE-induced phosphorylation of Hsp27 was completely suppressed by SB202190 (Fig. 1, H and I). In contrast, SCH772984 and SB202190 alone, or in combination, did not significantly reduce PE-induced phosphorylation of Creb (Fig. 1, *H* and *J*).

We then examined the expression levels of *cTnT* and *Anp* as markers of hypertrophic signaling. SCH772984 or SB202190 alone prevented the PE-induced *cTnT* expression and were most potent when used in combination (Fig. 1*K*). On the other hand, SCH772984, but not SB202190, significantly reduced PE-induced *Anp* (Fig. 1*L*), consistent with a regulatory pathway linking Erk1/2 activation to GATA-mediated transcription of *Anp* (64). Collectively, these experiments demonstrate that Erk1/2 and p38 cooperatively contribute to the growth response in NRVMs downstream of PE.

### Metabolic labeling with clickable Ac4GalNAlk identifies O-GlcNAc–modified versions of p38 and Erk1/2

**Figure 2:**
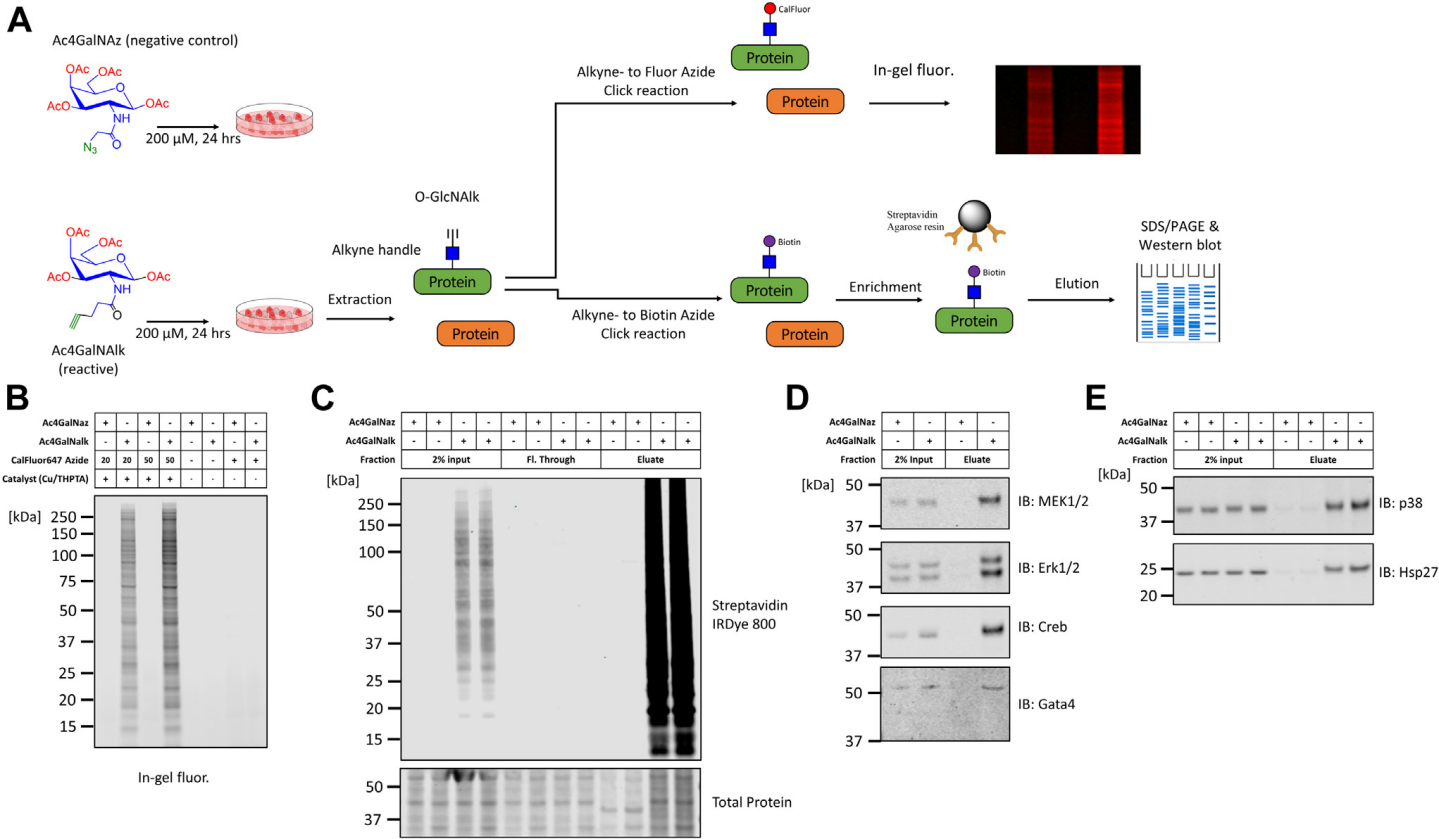
**Bio-orthogonal metabolic labeling of glycans coupled with enrichment and immunoblotting identifies O-GlcNAcylated members of the MAPK pathway.***A*, schematic of the experimental setup for the metabolic labeling of cells with the sugar analogs Ac4GalNAz (negative control) or Ac4GalNAlk. The latter serves as the alkyne donor in a copper-catalyzed alkyne-azide ‘click’ reaction. Cells were incubated with 200 μM Ac4GalNAz or Ac4GalNAlk for 24 h prior to protein extraction. Subsequently, protein extracts are subject to ‘click’ with CalFluor Azide and in-gel fluorescence scanning or Biotin Azide for streptavidin pull-down and Western blot detection. *B*, protein extracts (20 μg) were reacted with CalFluor 647 Azide (final concentrations 20 μM or 50 μM) with or without the copper catalyst. The samples were resolved on a gel and scanned to detect modified proteins by in-gel fluorescence. *C*, NRVMs metabolically labeled with Ac4GalNAz or Ac4GalNAlk for 24 h were collected and lysed. Five hundred micrograms of extracted protein were reacted with biotin azide and the ‘click’ reaction was cleaned-up by methanol/chloroform precipitation. Precipitated proteins were resuspended in 0.05% SDS, 50 mM Tris HCl, pH 8.0, and 2% of the input was taken before adding the protein suspension to streptavidin-agarose beads for binding (see also Experimental procedures for details). All collected fractions were loaded in duplicates and probed with streptavidin IR Dye 800 in Western blot. *D*, protein samples obtained after ‘click’ reaction onto biotin azide plus, enriched as described above and analyzed by immunoblotting for the presence of glycosylated and enriched candidates MEK1/2, Erk1/2, Creb, and Gata4. *E*, protein samples obtained as described above were analyzed by immunoblotting for the presence of glycosylated and enriched candidates p38 and Hsp27. The samples were loaded in duplicates in this experiment. MAPK, mitogen-activated protein kinase; NRVM, neonatal rat ventricular myocyte; O-GlcNAc, O-linked GlcNAc.

Next, we interrogated whether p38 and Erk1/2 were O-GlcNAcylated. To do that, we employed metabolic labeling in cells using the sugar analogs Ac4GalNAz or Ac4GalNAlk. These are biosynthetically converted into UDP-GalNAz or UDP-GalNAlk respectively, serving as substrates of OGT in intracellular protein O-GlcNAcylation (65, 66, 67) (Fig. 2*A*). In a proof of concept experiment, we treated cells with 200 μM Ac4GalNAz (negative control) or Ac4GalNAlk for 24 h, and protein extracts were used in Alkyne-Azide click reactions with CalFluor 647 azide, a probe that fluoresces only after it reacts with an alkyne (68). Using in-gel fluorescence, we detected widespread incorporation of the fluorogenic probe in proteins from Ac4GalNAlk-treated cells, but not in control Ac4GalNAz-treated cells (Fig. 2*B*). Furthermore, there was no background fluorescent reactivity in the absence of the click catalyst (Cu/THPTA, Fig. 2*B*). Next, we treated NRVMs with Ac4GalNAz or Ac4GalNAlk as above and proteins were extracted from metabolically labeled cells. Alkyne-azide click reactions were performed with biotin azide and biotinylated proteins were pulled-down using streptavidin-conjugated agarose beads. The different fractions from the pull-down were subjected to Western blot with streptavidin IR Dye 800 showing the efficient enrichment of biotinylated proteins with this approach (Fig. 2*C*).

We next analyzed the biotinylated samples to identify evidence for O-GlcNAcylation on MAPKs Erk1/2 and p38. Indeed, by Western blotting, we found that the pulled-down fraction contained Erk1/2 and also its upstream MAP2Ks, MEK1/2 (Fig. 2*D*). The MAPK-downstream transcription factor Creb was also detected, consistent with previous reports showing that it is O-GlcNAcylated (55). Another transcription factor regulated by MAPK signaling, Gata4, was also present in the pull-down (Fig. 2*D*). Moreover, we found that the other MAPK of interest, p38, was pulled-down and so was its downstream target Hsp27 (Fig. 2*E*). Taken together, these findings indicate that several members in the MAPK signaling pathway are putative targets for O-GlcNAcylation in cardiac myocytes.

### TMG and OSMI-1 respectively increase and decrease protein O-GlcNAcylation in NRVMs without impacting the overall abundance of other glycans

**Figure 3:**
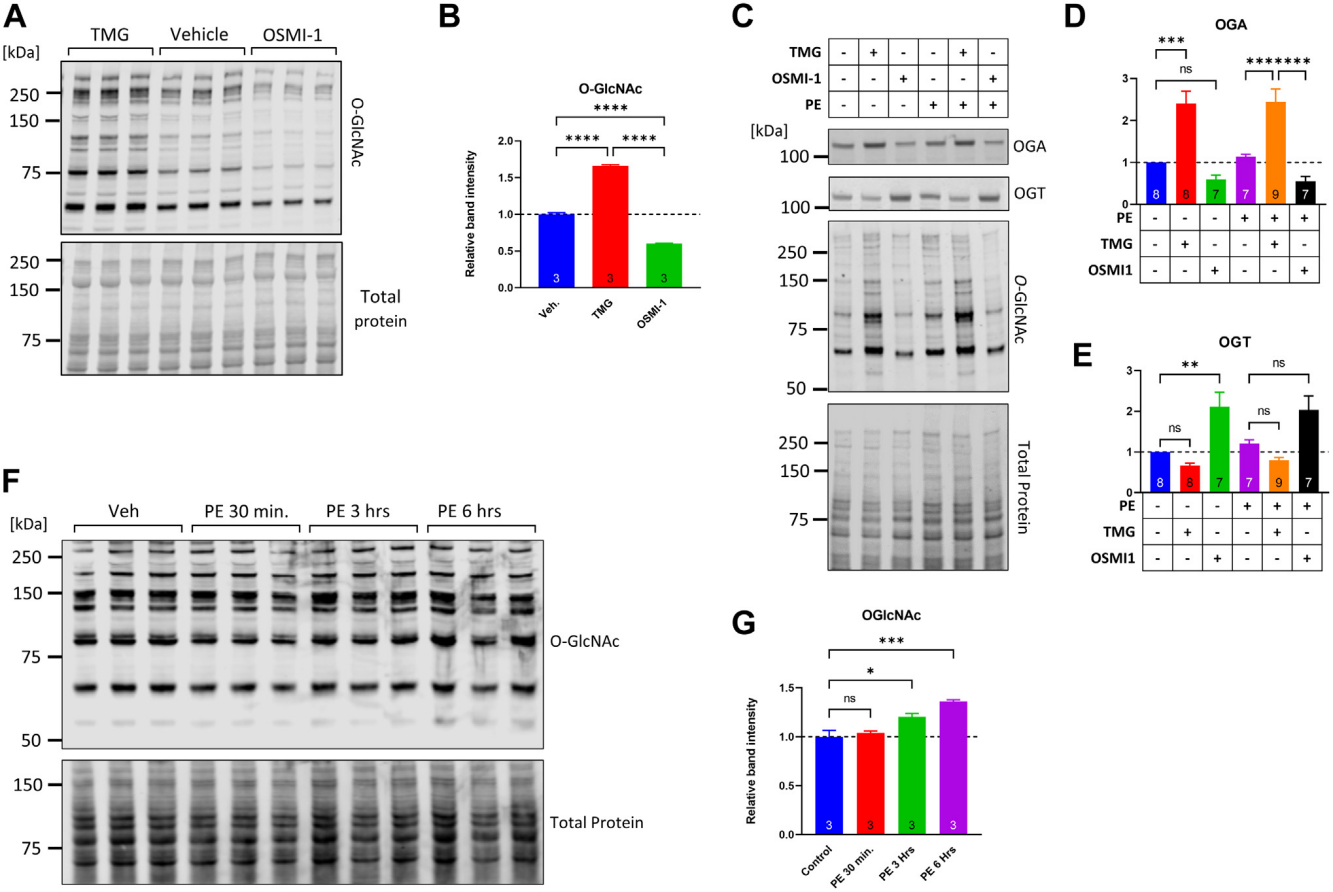
**TMG and OSMI-1, chemical inhibitors of OGA and OGT, respectively, increase and decrease protein O-GlcNAcylation with reciprocal changes in the protein abundance of OGA and OGT.***A* and *B*, NRVMs incubated for 24 h in medium without growth factors were exposed to 200 nM Thiamet G (TMG) or 25 μM OSMI-1 and were harvested 6 h later for Western blot analysis. The detection of O-GlcNAcylated proteins was carried out using antibody RL2 (mouse IgG), and the cumulative band signal along each individual lane ranging from 50 to 250 kDa was quantified and expressed relative to the total protein load. Summary results are shown in (*B*). Comparisons across treatment groups were done with one-way ANOVA and Tukey post hoc test, ∗∗∗∗*p* < 0.0001, n = 3. *C*–*E*, NRVMs were pretreated for 6 h with TMG or OSMI-1 and then exposed to 5 μM PE for an additional 30 min. Samples were analyzed for protein abundance of OGA, OGT, and overall protein O-GlcNAcylation. The bar graphs in (*D* and *E*) show the mean OGA and OGT abundance across the different treatment groups, and the number of biological replicates are indicated inside the bars. *F* and *G*, NRVMs incubated for 24 h in medium without serum were left untreated (Control) or treated with 5 μM PE for 30 min, 3 h, or 6 h after which they were harvested for O-GlcNAc Western blot analysis and quantification as described above. The summary of the quantifications is shown in (*G*). Comparisons across treatment groups were done with one-way ANOVA and Tukey post hoc test, ∗*p* < 0.05, ∗∗ *p*< 0.01, ∗∗∗*p* < 0.001. NRVM, neonatal rat ventricular myocyte; O-GlcNAc, O-linked GlcNAc; OGA, O-GlcNAcase; OGT, O-GlcNAc transferase; PE, phenylephrine.

Protein O-GlcNAcylation was manipulated in NRVMs with Thiamet G (TMG) and OSMI-1, inhibitors of OGA and OGT, respectively (69, 70). Treatment with TMG (200 nM, 6 h) led to more than 50% increase in protein O-GlcNAcylation, while OSMI-1 (25 μM) reduced O-GlcNAcylation by 50% (Fig. 3, *A* and *B*). Consistent with earlier reports, OGA protein was significantly upregulated in cells treated with TMG (2.4-fold higher, TMG *versus* vehicle, Fig. 3, *C* and *D*), whereas OGT was significantly upregulated in cells treated with OSMI-1 (2.1-fold higher, OSMI-1 *versus* vehicle, Fig. 3, *C* and *E*). PE treatment (5 μM, 30 min) did not significantly change the levels of O-GlcNAcylation, although longer treatment durations (3 and 6 h) induced modest but significant elevations in protein O-GlcNAcylation (20.5% and 36.3% increase respectively, Fig. 3, *F* and *G*).

Furthermore, we examined whether TMG or OSMI-1 altered the abundance of GlcNAc-, GalNAc-Galactose–, and GalNAc-Mannose–bearing glycoproteins, whose biosynthesis might be affected by potential alterations in UDP-GlcNAc levels caused by either TMG or OSMI-1. Lectin blots with biotinylated wheat germ agglutinin, peanut agglutinin, and *Dolichos biflorus* agglutinin did not identify changes in glycan abundances between control and TMG- or OSMI-1–treated cells (Fig. S1, *A*–*E*). Given OSMI-1’s structural similarity with UDP-GlcNAc, we examined whether OSMI-1 might elicit a confounding impact on N-linked glycosylation. However, lectin blots for Concanavalin A (ConA) revealed that the abundances of N-linked glycoproteins were not impacted significantly by OSMI-1 (Fig. S1, *F* and *G*). On the other hand, tunicamycin, which blocks the addition of GlcNAc onto dolichol phosphate, induced a significant reduction in the ConA signal (Fig. S1, *F* and *G*).

### OGT inhibition with OSMI-1 induces p38 phosphorylation and downstream signaling

**Figure 4:**
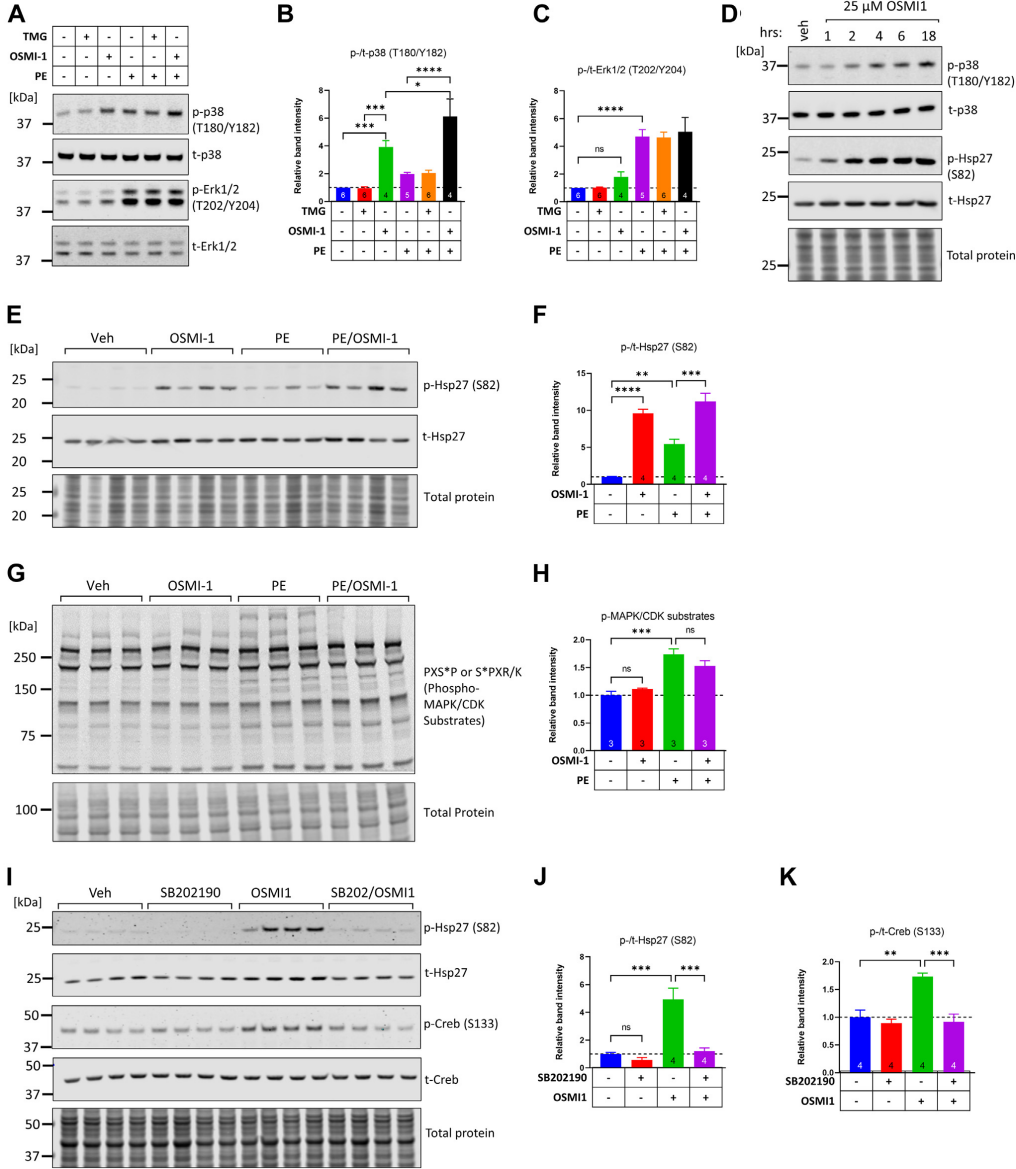
**OGT inhibition induces phosphorylation of p38 and activates its downstream signaling pathway.***A*–*C*, representative western blots of p38 and Erk1/2 (phospho-specific and total). Cells were exposed for 6 h to TMG (200 nM), OSMI-1 (25 μM), or a combination of TMG/OSMI-1, and this was followed by treatment for 30 min with or without PE (5 μM). Comparisons across the different samples were performed with one-way ANOVA and Tukey post hoc test. ∗*p* < 0.05, ∗∗∗*p* < 0.001, ∗∗∗∗*p* < 0.0001, ns; not significant (*p* = 0.88). The number of biological replicates in each group is indicated in their respective bars. *D*, Western blot analysis of p38 and Hsp27 phosphorylation during a time-course of 1 to 18 h of exposure to OSMI-1. *E* and *F*, Western blot and quantitation of Hsp27 phosphorylation induced by 25 μM OSMI-1 (6 h), 5 μM PE (30 min), or both. Comparisons between groups were done with one-way ANOVA and Tukey post hoc test. ∗∗*p* < 0.01, ∗∗∗*p* < 0.001, ∗∗∗∗*p* < 0.0001, four biological replicates per group. *G* and *H*, Western blot of proteins with the phosphorylated motifs PXSP or SPXR/K, target sites of members of the MAPK, and cyclin dependent kinases (CDKs). For quantifications, the cumulative band intensities along the lane were obtained from proteins with molecular weights of 50 to 250 kDa and were normalized with the respective cumulative lane intensities from the total protein blots. Comparisons between groups were done with one-way ANOVA and Tukey post hoc test. ∗∗∗*p* < 0.001, the comparison between PE *versus* PE/OSMI-1 yields *p* = 0.57, n = 3 biological replicates per group. *I*–*K*, Western blot and quantitation of Hsp27 and Creb phosphorylation induced by 25 μM OSMI-1 (6 h) but repressed by the p38 inhibitor SB202190 (10 μM, 6 h). Comparisons between groups were done with one-way ANOVA and Tukey post hoc test. ∗∗∗*p* < 0.001, ∗∗∗∗*p* < 0.0001, four biological replicates per group. MAPK, mitogen-activated protein kinase; OGT, O-GlcNAc transferase; PE, phenylephrine; TMG, Thiamet G.

Next, we examined the effects of TMG and OSMI-1 on MAPK phosphorylation with or without PE. We found that OSMI-1 induced a nearly 4-fold increase in p38 phosphorylation (3.9-fold increase *versus* untreated, *p* < 0.001, Fig. 4, *A* and *B*). In contrast, TMG alone or in combination with PE did not impact p38 phosphorylation. Moreover, OSMI-1 had no effect on Erk1/2 phosphorylation, either at baseline or in PE-treated conditions (Fig. 4, *A* and *C*). p38 phosphorylation gradually increased during an 18-h exposure to OSMI-1, mirrored by a progressive increase in Hsp27 phosphorylation (Fig. 4*D*). Hsp27 phosphorylation was increased in PE-stimulated conditions, although OSMI-1 alone caused higher Hsp27 phosphorylation (Fig. 4, *E* and *F*). We next examined if OSMI-1 caused widespread changes in the phosphorylation of MAPK substrates. While we found that phosphorylation of MAPK/CDK substrates was increased by PE treatment, this was not the case in OSMI-1–treated cells (Fig. 4, *G* and *H*). These data suggest that the induction of p38 phosphorylation by OSMI-1 is a selective event that impacts the MAPK signaling pathway at a level that is distinct from alpha-1 adrenergic activation and the broad induction of its downstream effectors (*e.g.*, phospholipase C, inositol triphosphate, diacylglycerol, Ca^2+^). Finally, the p38 inhibitor SB202190 was sufficient to completely abolish the OSMI-1–induced phosphorylation of Hsp27 and it was also effective at decreasing the OSMI-1–induced phosphorylation of the transcription factor Creb (Fig. 4, I–*K*). Collectively, these experiments indicate that OGT inhibition selectively activates p38 phosphorylation and its downstream signaling.

**Figure 5:**
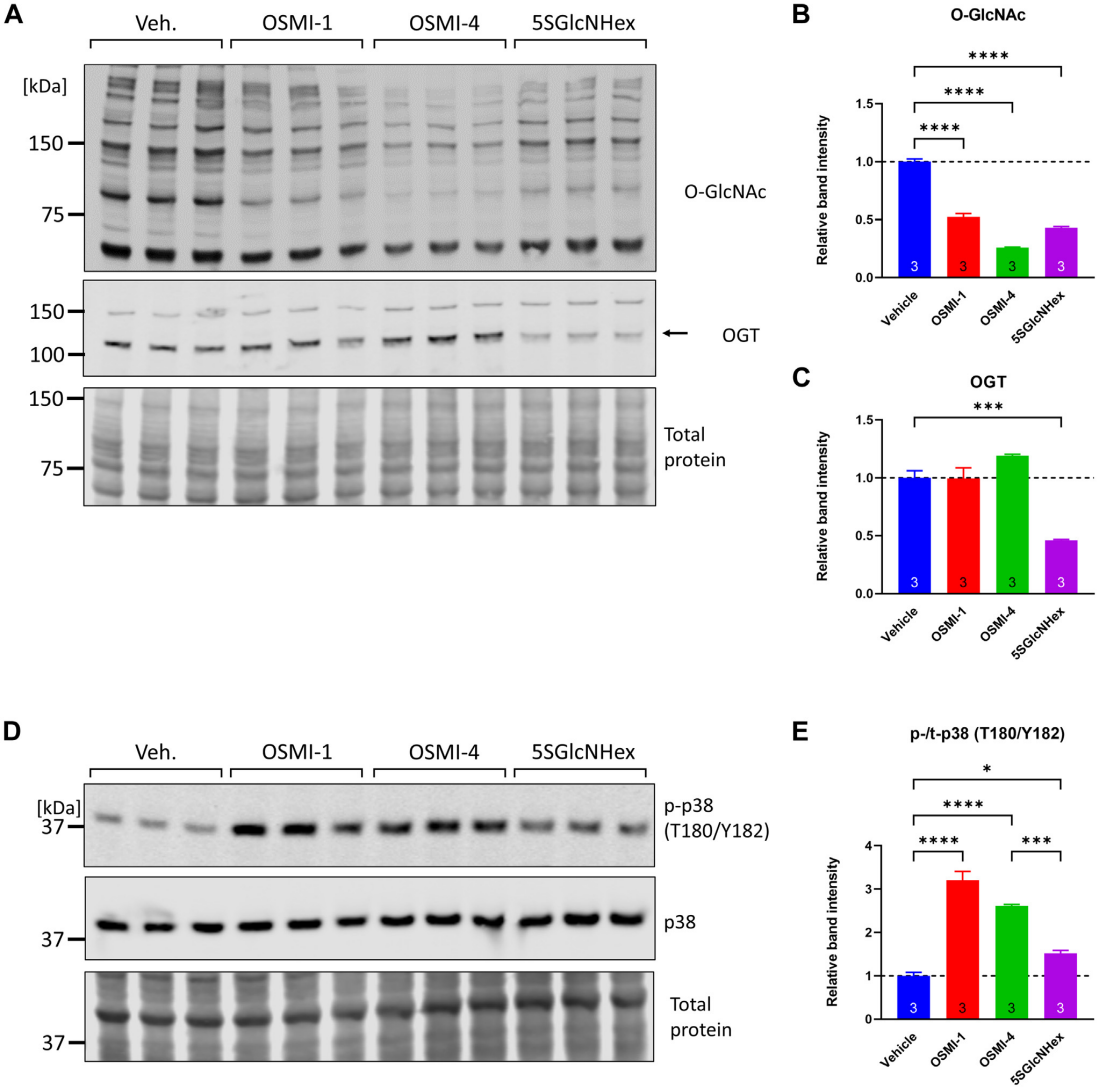
**Three different OGT inhibitors lower protein O-GlcNAcylation and induce p38 phosphorylation in NRVMs.***A*–*C*, NRVMs were treated with the OGT inhibitor OSMI-1 (25 μM), its structurally related derivative OSMI-4 (25 μM, ethylester form -4b), and the precursor metabolic inhibitor 5SGlcNHex (25 μM). After 6 h of treatment, cell extracts were analyzed for changes in protein O-GlcNAcylation and OGT protein abundance. Statistical differences between groups were assessed by one-way ANOVA and Tukey post hoc test. ∗∗∗*p* < 0.001, ∗∗∗∗*p* < 0.0001, three biological replicates per group. *D* and *E*, NRVM extracts obtained as described above were analyzed for p38 phosphorylation. Statistical differences between groups were assessed by one-way ANOVA and Tukey post hoc test. ∗*p* < 0.05, ∗∗∗*p* < 0.001, ∗∗∗∗*p* < 0.0001, three biological replicates per group. NRVM, neonatal rat ventricular myocyte; O-GlcNAc, O-linked GlcNAc; OGT, O-GlcNAc transferase.

Following the finding that reducing protein O-GlcNAcylation with OSMI-1 triggers p38 phosphorylation, we tested if other OGT inhibitors induce p38 phosphorylation. OSMI-4 is structurally related to OSMI-1 and inhibits OGT by competitive docking into the UDP-GlcNAc binding pocket of OGT (71). In addition, we used 5SGlcNHex, a metabolic inhibitor that is converted to UDP-5SGlcNAc which then functions as a competitive OGT inhibitor (72). Treating NRVMs with each inhibitor (25 μM, 6 h) induced significant reductions in protein O-GlcNAcylation (52.2%, 25.9%, and 42.9% of baseline for OSMI-1, OSMI-4, and 5SGlcNHex, respectively; Fig. 5, *A* and *B*). Importantly, all three inhibitors induced significant increases in p38 phosphorylation (3.4-fold, 3.0-fold, and 1.5-fold increases for OSMI-1, OSMI-4, and 5SGlcNHex, respectively; Fig. 5, *D* and *E*), confirming that the effect was not an epiphenomenon of the particular chemical structure of OSMI-1.

### OGT inhibition with OSMI-1 prevents the O-GlcNAcylation of p38

As shown earlier, using metabolic labeling with Ac4GalNAlk, we found evidence that p38 is directly O-GlcNAcylated (Fig. 2*E*). Given OSMI-1’s impact on potently increasing p38 phosphorylation, we examined whether OSMI-1 was directly affecting p38 O-GlcNAcylation. To that end, we metabolically labeled HEK293 cells with Ac4GalNAlk, concomitantly with exposure to vehicle, TMG, or OSMI-1 (see also schematic in Fig. S2*A*). Using streptavidin-mediated pull-down, we found efficient enrichment of biotinylated proteins in Ac4GalNAlk-treated cells but not in Ac4GalNAz negative control cells (Fig. S2*B*). Follow-up Western blots showed that p38 was present in pull-downs from vehicle- and TMG-treated cells, but not in OSMI-1–treated cells (Fig. S2*C*). These findings further confirm the previous observations that p38 is O-GlcNAcylated at baseline and demonstrate that this can be prevented by OGT inhibition with OSMI-1.

### Activation of p38 downstream of OSMI-1 is dependent on canonical MAP3K–MAP2K signaling axis

**Figure 6:**
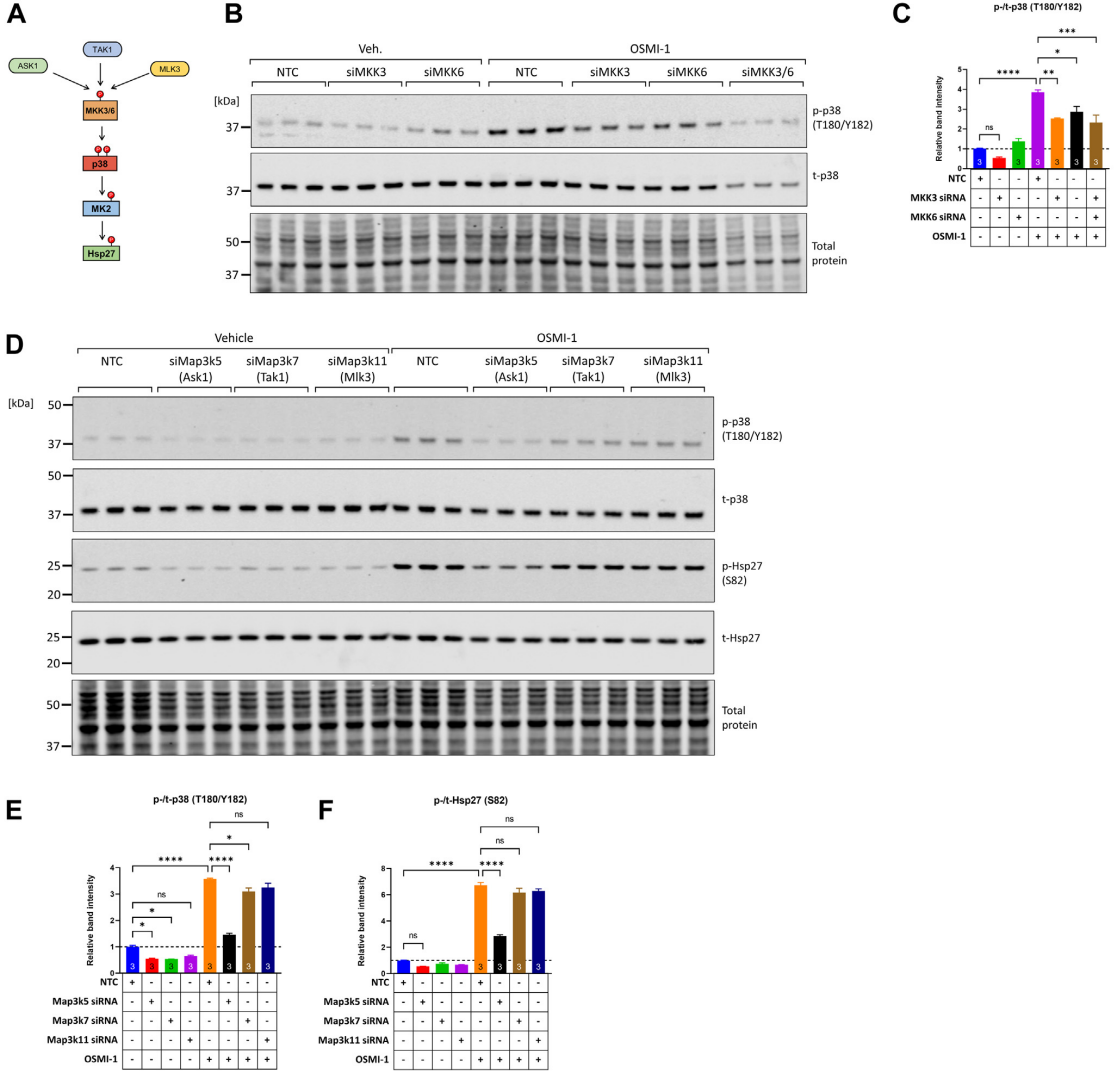
**Phosphorylation of p38 downstream of OGT inhibition is mediated by MAP2Ks MKK3/6 and MAP3K Ask1.***A*, schematic illustrating the canonical pathway of p38 phosphorylation involving MAP3Ks Ask1, Tak1, Mlk3, and MAP2Ks MKK3 and MKK6. *B* and *C*, NRVMs were transfected with nontargeting control (NTC), MKK3-, or MKK6-targeting siRNA (alone or combined, final concentration 20 nM) and 48 h later, they were treated with vehicle or OSMI-1 (25 μM) for an additional 6 h. Subsequently, cell extracts were analyzed for the phosphorylation of p38 by Western blot. *D*–*F*, NRVMs were transfected with NTC, Map3k5 (Ask1)-, Map3k7 (Tak1)-, or Map3k11 (Mlk3)-targeting siRNAs (final concentration 20 nM) and 48 h later, they were treated with vehicle or OSMI-1 (25 μM) for an additional 6 h. Subsequently, cell extracts were analyzed for the phosphorylation of p38 and Hsp27 by Western blot. Statistical differences were assessed by two-way ANOVA and Tukey post hoc test. ∗*p* < 0.05, ∗∗*p* < 0.01, ∗∗∗*p* < 0.001, ∗∗∗∗*p* < 0.0001, ns; not significantly different. NRVM, neonatal rat ventricular myocyte; OGT, O-GlcNAc transferase.

Next, we examined whether upstream kinases might be involved in the phosphorylation of p38. There are currently very few commercially available inhibitors for MKK3 and MKK6, which are the immediate upstream activators of canonical p38 phosphorylation (see also schematic in Fig. 6*A*). Therefore, we employed siRNA-mediated knockdown of MKK3 and MKK6, to test whether their depletion could impair the observed OSMI-1–induced p38 phosphorylation. Indeed, we found that transfecting NRVMs with siRNAs targeting MKK3 or MKK6 significantly decreased OSMI-1–induced phosphorylation of p38 (34.7% reduction, OSMI-1/nontargeting control siRNA *versus* OSMI-1/siMKK3 and 25.9% reduction, OSMI-1/nontargeting control siRNA *versus* OSMI-1/siMKK6, *p* < 0.05, Fig. 6, *B* and *C*).

MAP3Ks Ask1, Tak1, and Mlk3 are well-known inducers of MKK3/6 in canonical p38 activation and have been described as potential MKK3/6 activators in cardiac myocytes (Fig. 6*A*). The Ask1-specific inhibitor GS-444217 was tested for its potency to abolish OSMI-1–induced p38 phosphorylation (1 μM GS-444217 used alone or together with OSMI-1 for 6 h treatment). However, we observed that this treatment did not significantly reduce OSMI-1–induced p38 phosphorylation or the phosphorylation of downstream Hsp27 (Fig. S3, *A*–*C*). Consistently, when a range of GS-444217 concentrations was used (100 nM, 500 nM, 1 μM), we did not observe reductions in OSMI-1–induced p38 phosphorylation, and when a higher concentration of GS-444217 was used (10 μM), it potentiated instead of inhibiting OSMI-1–induced p38 phosphorylation and downstream Hsp27 phosphorylation (Fig. S3, *D*–*F*). Next, we used the Tak1-specific inhibitor Takinib, either alone or in combination with GS-444217, and examined whether this treatment could impair OSMI-1–induced p38 phosphorylation. However, OSMI-1–induced p38 phosphorylation was still observed in the presence of Takinib, and it was further potentiated by the presence of GS-444217 (Fig. S3, *G* and *H*). Finally, we examined the effect of Mlk3 inhibitor URMC-099. Unexpectedly, this inhibitor was potent in inducing p38 alone (Fig. S3, *I* and *J*), making it unsuitable for the study of the OSMI-1–induced p38 phosphorylation mechanism. It is unclear why GS-444217 or URMC-099 induces p38 phosphorylation. One potential explanation could be due to off-target effects on other kinases operating upstream of p38 phosphorylation. Another possibility could be that accumulation of the kinase (*e.g.*, Ask1) in its inactive state in the cell elicits secondary pathways leading to the paradoxical p38 phosphorylation and activation.

Given these drawbacks with the MAP3K inhibitors, we resorted to siRNA-mediated knockdown of the kinases. Importantly, we found that transfecting NRVMs with an Ask1-targeting siRNA induced a significant reduction in OSMI-1–induced p38 phosphorylation (Fig. 6, *D* and *E*). A modest reduction in OSMI-1–induced p38 phosphorylation was also observed with Tak1-targeting siRNA, whereas targeting Mlk3 did not reach statistical significance (Fig. 6, *D* and *E*). Importantly, the reduction in p38 phosphorylation by Ask1-targeting siRNA led to a significant reduction in downstream Hsp27 phosphorylation (Fig. 6*F*).

Taken together, these findings with siRNA-mediated MAP3K targeting illustrate that OSMI-1–induced p38 phosphorylation is mediated by MKK3/6 MAP2Ks that are in turn activated predominantly by Ask1 with potentially lesser inputs from Tak1 MAP3K.

### OSMI-1–induced p38 phosphorylation requires NADPH oxidase 2 subunits p47phox and gp91phox

**Figure 7:**
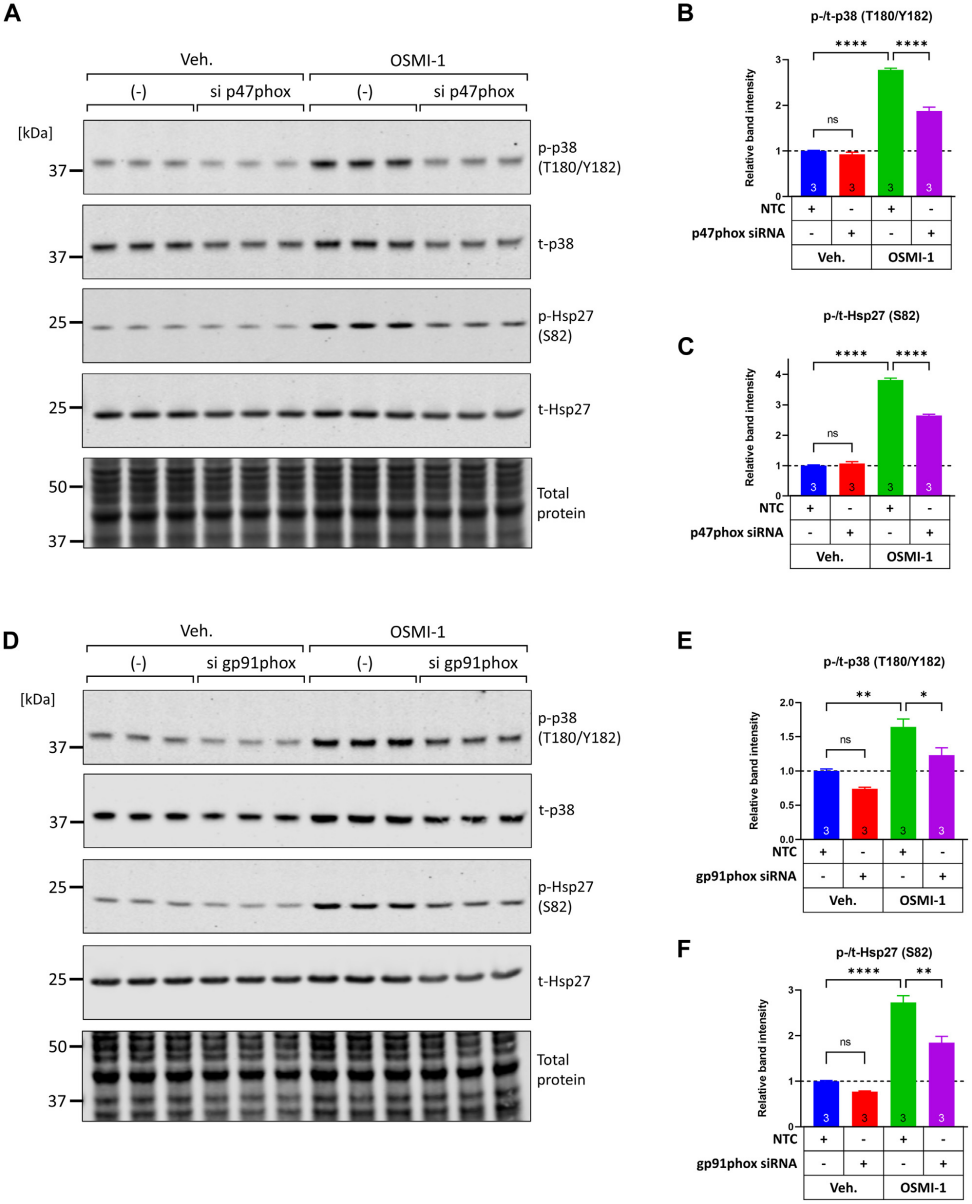
**Phosphorylation of p38 downstream of OGT inhibition requires NADPH oxidase 2 subunits p47phox and gp91phox.***A*–*C*, NRVMs were transfected with nontargeting control (NTC) or p47phox-targeting siRNA (the protein p47phox is encoded by the gene neutrophil cytosolic factor 1, *Ncf1*). After 48 h of transfection, cells were treated with vehicle or OSMI-1 (25 μM) for an additional 6 h. Cell extracts were analyzed for the phosphorylation of p38 and its downstream target Hsp27 by Western blot. *D*-*F*, NRVMs were transfected with NTC or gp91phox-targeting siRNA (the protein gp91phox, also referred to as Nox2 is encoded by the gene cytochrome B-245 beta chain, *Cybb*). After 48 h of transfection, cells were treated with vehicle or OSMI-1 (25 μM) for an additional 6 h and the cell extracts were analyzed for the phosphorylation of p38 and Hsp27. Statistical differences between groups were assessed by two-way ANOVA and Tukey post hoc test. ∗*p* < 0.05, ∗∗*p* < 0.01, ∗∗∗∗*p* < 0.0001, three biological replicates per group. NRVM, neonatal rat ventricular myocyte; OGT, O-GlcNAc transferase.

Ask1 can be activated due to the accumulation of reactive oxygen species (ROS) in the cytosol. One source of cytosolic ROS in cardiomyocytes is the family of NADPH oxidases (NOX), multicomponent enzymes on the plasma membrane that transfer electrons from NADPH to oxygen leading to superoxide production (73). To examine whether NOXs are implicated in OSMI-1–induced p38 activation in cardiomyocytes, we used siRNA to target p47phox (encoded by gene neutrophil cytosolic factor 1, *Ncf1*) a key regulatory subunit that upon activation translocates from the cytosol to the plasma membrane to associate with NOX2 and activate the production of superoxide. In agreement with a role in this pathway, we found that targeting p47phox significantly reduced the phosphorylation of p38 as well as the phosphorylation of downstream target Hsp27 in OSMI-1–treated cells (Fig. 7, *A*–*C*). Furthermore, we used siRNA to target the core subunit of NOX2, gp91phox. Similarly, this approach reduced the phosphorylation of p38 and Hsp27 (Fig. 7, *D*–*F*), further implicating NOX2 as a causal upstream mediator of OSMI-1–induced p38 phosphorylation through the canonical Ask1–MKK3/6 pathway.

### Activation of p38 downstream of OSMI-1 is dependent on the noncanonical signaling axis and requires Tab1

**Figure 8:**
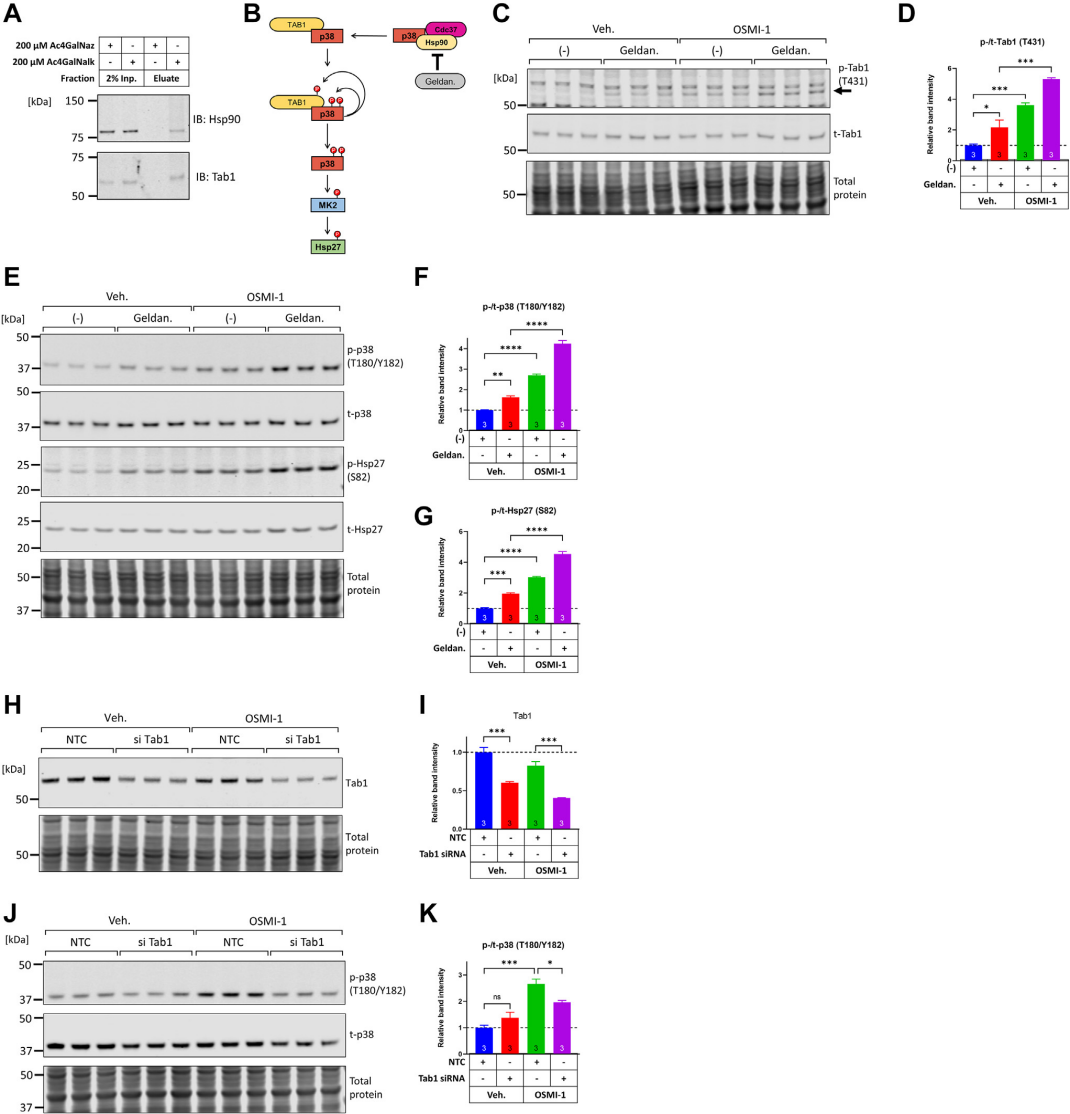
**Phosphorylation of p38 downstream of OGT inhibition is mediated by the noncanonical p38 activation pathway and requires protein Tab1.***A*, protein samples obtained after ‘click’ reaction and enriched with streptavidin pull-down were analyzed by immunoblotting for the presence of glycosylated targets Hsp90 and Tab1. *B*, schematic illustrating the noncanonical pathway for p38 activation, including the inhibitory complex Hsp90/Cdc37 and the scaffold protein Tab1. The Hsp90 inhibitor geldanamycin is also shown. *C*–*G*, NRVMs were treated with vehicle or OSMI-1 (25 μM) for 5 h, and then vehicle or geldanamycin (2 μM) were added to the cells for the remaining 1 h of incubation. Protein extracts were analyzed for the phosphorylation of Tab1, p38, and Hsp27 across the different treatment groups. Statistical differences between treatment groups were assessed by one-way ANOVA and Tukey post hoc test. ∗*p* < 0.05, ∗∗*p* < 0.01, ∗∗∗*p* < 0.001, ∗∗∗∗*p* < 0.0001. *H*–*K*, NRVMs were transfected with nontargeting control (NTC) or Tab1-targeting siRNA (final concentration 20 nM) and 48 h later, they were treated with vehicle or OSMI-1 (25 μM) for an additional 6 h. Cell extracts were analyzed for Tab1 protein knockdown and phosphorylation of p38. Statistical differences between treatment groups were assessed by two-way ANOVA and Tukey post hoc test. ∗*p* < 0.05, ∗∗*p* < 0.01, ∗∗∗*p* < 0.001, ∗∗∗∗*p* < 0.0001, ns; not significantly different. NRVM, neonatal rat ventricular myocyte; OGT, O-GlcNAc transferase.

In addition to the canonical activation of p38 by MAP3Ks, p38 can undergo noncanonical activation by autophosphorylation. p38’s autophosphorylation in cardiac myocytes is kept in check by the inhibitory complex Hsp90/Cdc37, which sequesters p38 from interacting with its activating scaffold Tab1 (74, 75). Additionally, Tab1 is known to undergo O-GlcNAcylation (76). In agreement, using metabolic labeling with Ac4GalNAlk in cardiac myocytes, we found that both Hsp90 and Tab1 were O-GlcNAcylated (Fig. 8*A*). These findings indicated that the noncanonical pathway of p38 activation (see also schematic in Fig. 8*B*) was potentially subject to regulation by OSMI-1. To address that, we first examined whether the Hsp90 inhibitor geldanamycin could induce noncanonical p38 signaling in NRVMs. Indeed, geldanamycin alone increased the phosphorylation of Tab1, and this effect was additive to the induction of Tab1 phosphorylation caused by OSMI-1 (Fig. 8, *C* and *D*). Similar to Tab1, geldanamycin increased the phosphorylation of p38 and Hsp27 in a manner that was additive to the effect of OSMI-1 (Fig. 8, *E*–*G*). These findings indicate that OSMI-1 is not an inhibitor of Hsp90 *per se* but rather operates parallel to Hsp90 inhibition to further increase p38 autophosphorylation.

To further investigate the role of the noncanonical autophosphorylation pathway in OSMI-1–induced p38 phosphorylation, we knocked down Tab1 in NRVMs with siRNA. This approach led to a significant reduction in total Tab1 protein levels by 50 to 55% in the absence or presence of OSMI-1 (Fig. 8, H and I). Notably, knockdown of Tab1 decreased the OSMI-1–induced phosphorylation of p38 (23.4% reduction, OSMI-1 *versus* OSMI-1/Tab1 si, *p* < 0.05, Fig. 8, *J* and *K*), implicating Tab1 as an additional mediator of p38 phosphorylation in OGT-inhibited NRVMs.

### OGT inhibition with OSMI-1 perturbs PE-induced Erk1/2 and p38 signaling and impairs PE-induced hypertrophic growth of cardiac myocytes

**Figure 9:**
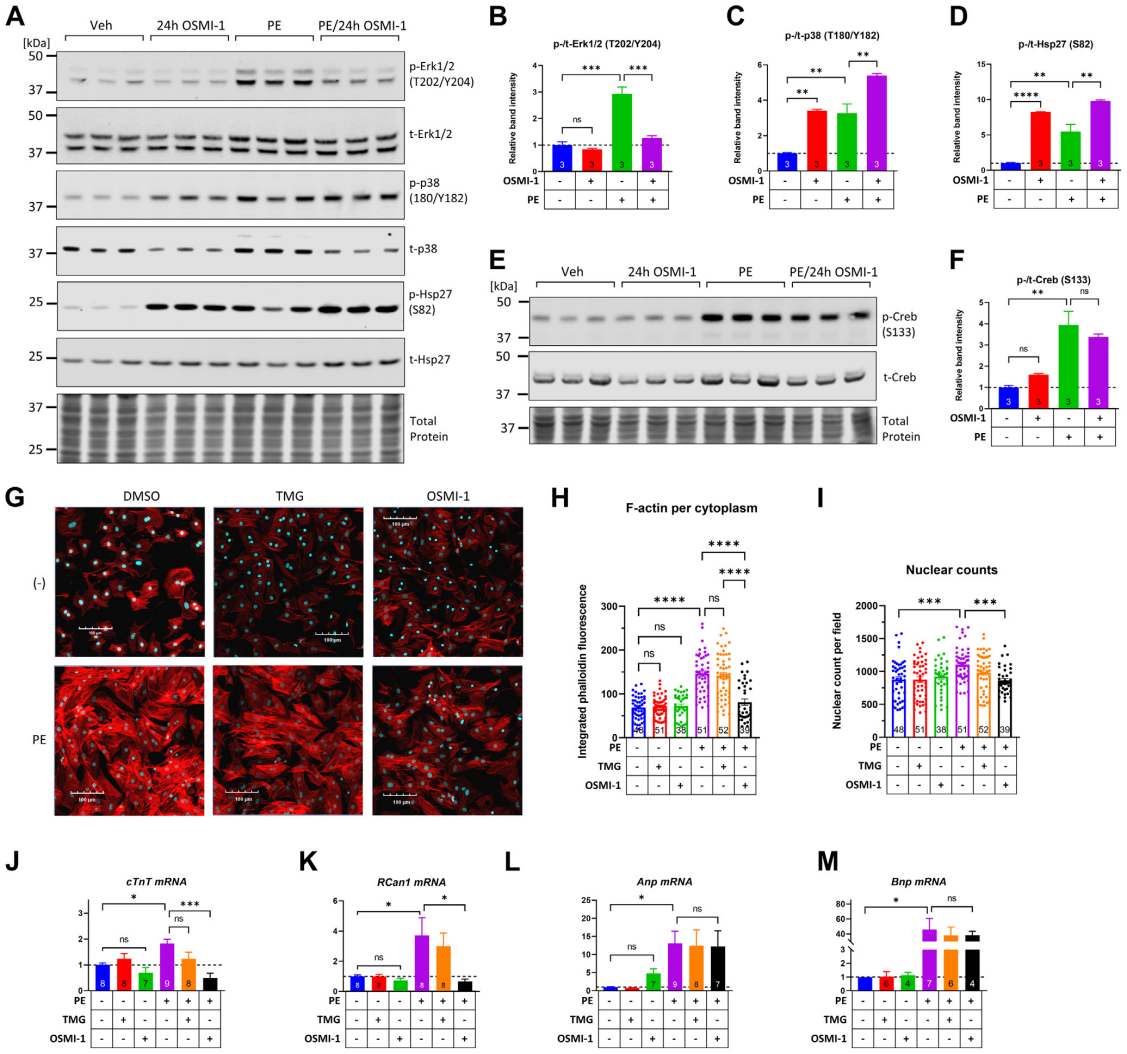
**OGT inhibition disrupts Erk1/2 and p38 phosphorylation and impairs the hypertrophic growth of NRVMs.***A*–*F*, Western blot and quantitation of Erk1/2, p38, and Hsp27 phosphorylation at 24 h after OSMI-1 exposure. NRVMs were treated with OSMI-1 (25 μM) and 24 h later, they were exposed to PE (5 μM) for an additional 30 min to stimulate signaling. Protein extracts were then analyzed for the phosphorylation of Erk1/2, p38, Hsp27, and Creb across the different treatment groups. *G*, NRVMs were treated with the OGA inhibitor TMG (200 nM), the OGT inhibitor OSMI-1 (25 μM), or both and 6 h later, they were exposed to PE (5 μM). After 24 h of combined treatment, the cells were fixed and stained with phalloidin-Alexa 594 to detect and quantify F-actin by confocal microscopy. The scale bar represents 100 μm. *H* and *I*, F-actin signal intensity per cytoplasm and total number of nuclei identified per field of view. The number of fields quantified per treatment group is shown in the respective bar graphs. Bars represent means ± standard error. Comparisons across treatment groups were done with one-way ANOVA and Tukey post hoc test. ∗∗∗*p* < 0.001, ∗∗∗∗*p* < 0.0001; ns; not significantly different. *J*–*M*, cTnT, RCan1, Anp, and Bnp mRNA expression across the indicated treatment groups. Cells were pretreated with TMG (200 nM), OSMI-1 (25 μM), or both, and 6 h later, they were exposed to PE (5 μM) for 24 h followed by RNA isolation and cDNA synthesis for quantitative PCR. The number of biological replicates in each treatment group are shown in their respective bars. Bars represent means ± standard error. Comparisons across treatment groups were done with one-way ANOVA and Tukey post hoc test. ∗*p* < 0.05, ∗∗*p* < 0.01, ∗∗∗*p* < 0.001, ∗∗∗∗*p* < 0.0001, ns; not significantly different. NRVM, neonatal rat ventricular myocyte; OGA, O-GlcNAcase; OGT, O-GlcNAc transferase; PE, phenylephrine; TMG, Thiamet G.

Next, we examined the impact of prolonged OSMI-1 treatment on the PE-induced effects on Erk1/2 and p38 phosphorylation. PE-induced phosphorylation of prohypertrophic Erk1/2 was significantly blunted by the 24-h OSMI-1 treatment (Fig. 9, *A* and *B*). In contrast to reducing the phosphorylation of Erk1/2, treatment with OSMI-1 for 24 h further increased the phosphorylation of pathologic p38 (Fig. 9, *A* and *C*). Interestingly, the increase in relative p38 phosphorylation was in part due to decreased protein levels of total p38 (Fig. 9*A*), suggesting perhaps the recruitment of compensatory mechanisms to restrict further pathologic increase in p38 activity. However, the phosphorylation of the p38-downstream factor Hsp27 was also significantly elevated in OSMI-1–treated cells, consistent with increased p38 activity (Fig. 9, *A* and *D*). On the other hand, the 24-h OSMI-1 treatment had no significant effect on basal Creb phosphorylation and also did not impact its PE-induced phosphorylation (Fig. 9, *E* and *F*).

Given the increase in protein abundance of OGT observed after a 6-h OSMI-1 treatment (Fig. 3*E*), we examined whether the same was true for the 24-h treatment. Indeed, OSMI-1 significantly increased the protein levels of OGT and reciprocally decreased the protein levels of OGA (Fig. S4, *A*–*C*).

Finally, we examined whether manipulating O-GlcNAcylation could impact PE-induced NRVM hypertrophy. Cells were pretreated with OSMI-1 or TMG for 6 h followed by concomitant treatment with PE (5 μM, 24 h). PE induced a 2-fold increase in cell size in vehicle- and TMG-treated cells but remarkably, OSMI-1 prevented this hypertrophic growth (Fig. 9, *G* and *H*). This is consistent with the perturbed Erk1/2 and p38 signaling noted above. Furthermore, assessment of total nuclei numbers showed that OSMI-1, combined with PE, was associated with lower numbers (Fig. 9*I*), indicating that in addition to its growth-arresting effect, OSMI-1 also impairs myocyte viability in PE-stimulated conditions. At the molecular level, OSMI-1 suppressed the PE-induced increase of prohypertrophic genes *cTnT* and *RCan1* (Fig. 9, *J* and *K*) but still allowed the induction of pathologic markers *Anp* and *Bnp* (Fig. 9, *L* and *M*).

## Discussion

A close relationship between phosphorylation signaling cascades and O-GlcNAcylation has been previously recognized (26, 77). The MAPK signaling cascade is a key determinant of cardiomyocyte growth and it also confers myocyte adaptation to diverse cardiac stresses. In this work, we investigated the functional intersections between O-GlcNAcylation and MAPK signaling in cardiac myocytes at baseline and during physiological hypertrophic growth. The salient findings of the study are as follows: (1) the MAPKs Erk1/2 strongly contribute to the hypertrophic growth of cardiac myocytes downstream of PE stimulation, with lesser input from p38, (2) a subset of both Erk1/2 and p38 are O-GlcNAcylated, as well as signaling mediators upstream and downstream of the Erk1/2 and p38 kinases, (3) the OGT inhibitor OSMI-1 strongly and specifically reduces O-GlcNAcylation in cardiac myocytes and promotes a robust induction of p38 phosphorylation at early time points, and (4) prolonged exposure to OSMI-1 prevents PE-induced Erk1/2 phosphorylation, exacerbates p38 phosphorylation, and blunts the PE-induced growth of cardiomyocytes. Focusing specifically on the activation of p38 by OSMI-1, our findings suggest three potential mechanisms that are not mutually exclusive: (i) activation of a novel signaling axis involving NOX2/Ask1 that activates the canonical MAP2Ks MKK3/6 to phosphorylate and activate p38, (ii) activation of the noncanonical p38 autoactivation pathway that requires the scaffold protein Tab1, and (iii) a direct O-GlcNAcylation of p38 that could affect its phosphorylation.

Previously, it was found that cardiac myocytes exposed to high glucose underwent increased O-GlcNAcylation, associated with increased phosphorylation of Erk1/2 (78). A positive correlation between O-GlcNAc levels and Erk1/2 phosphorylation was also noted in gastric cancer cells and hippocampal slices (79, 80). Our findings on Erk1/2 phosphorylation during global O-GlcNAc manipulation suggest a more nuanced relationship. Firstly, we found that elevating global O-GlcNAcylation with TMG does not impact basal and PE-induced Erk1/2 phosphorylation and the same is true for a 6-h treatment with OSMI-1. However, we found the PE-induced Erk1/2 phosphorylation to be significantly blunted in cells exposed to OSMI-1 for 24 h. A search of proteomics compendia (81) yields hits for O-GlcNAcylation on Erk1/2 and the upstream kinases MEK1/2. In agreement with this bioinformatic information, we have provided evidence with metabolic labeling, pull-down, and direct Western blotting, that subsets of both Erk1/2 and MEK1/2 are O-GlcNAcylated. It remains unknown, however, how the loss of O-GlcNAcylation on these targets might lead to reduced Erk1/2 phosphorylation. One possibility is that O-GlcNAcylation prevents the phosphorylation on inhibitory T394 of MEK2 leading to more activation of Erk1/2 (82). Regardless of the underlying mechanism, OSMI-1–induced prevention of Erk1/2 phosphorylation is likely to play a major role in the blunted hypertrophic response we observe, because Erk1/2 is the primary signaling MAPK that drives cardiomyocyte growth during PE stimulation.

Concerning the other MAPK of interest, p38, we found that OSMI-1 was a potent inducer of its phosphorylation and activity, both at early and late time points (6 h and 24 h). Importantly, OSMI-1 induced the phosphorylation of p38 in basally treated cells and it also had an additive effect on p38 phosphorylation when cells were treated with PE. Thus, decreasing O-GlcNAcylation in cardiomyocytes correlates with increased p38 phosphorylation. This is in contrast with previous work in leukocytes and glomerular mesangial cells, where it was found that increasing O-GlcNAcylation (high glucose, treatment with increased glucosamine, PUGNAc, or TMG) potentiates rather than decreasing the phosphorylation of p38 (83, 84). These studies examined time points from 90 min up to 24 h of O-GlcNAc manipulation, which are similar to our treatments. However, because OSMI-1 was not used in these studies, it is difficult to make direct comparisons as to what might be accounting for the different outcomes in these different cell types. As for the upstream mechanisms driving p38 phosphorylation with increasing O-GlcNAcylation, the first study found increased activity of MKK3/6 (84), while the other found evidence for Ask1’s participation in p38 phosphorylation (83). In our work with cardiomyocytes, although the directionality of the manipulation is opposite (*i.e.*, reducing O-GlcNAcylation increases p38 phosphorylation), we found that the same pathway was involved (*i.e.*, Ask1/MKK3/6 leading to p38 phosphorylation).

Regulation of p38 phosphorylation by Ask1 in the context of ROS has been recently demonstrated in cardiac myocytes and hearts (85). Upstream activators of Ask1 are the ROS-generating NOXs and ablation of p47phox, the regulatory subunit of NOX2, ameliorates Ask1 phosphorylation and cardiac damage due to angiotensin-II infusion (86). Furthermore, excessively high levels of O-GlcNAc in cardiomyocytes (*e.g.*, due to hyperglycemia) lead to ROS production by NOX2 in cardiomyocytes (87, 88) and vascular smooth muscle cells (89). Along these lines, we investigated the implications of NOX2 in OSMI-1–induced p38 phosphorylation and found that p47phox and gp91phox, both subunits of NOX2 are required for p38 phosphorylation in OSMI-1–treated cells. There is currently a scarcity of studies identifying O-GlcNAcylation on one or more NOX2 subunits (gp91phox, p67phox, p47phox, p40phox, p22phox, or Rac1/2), so it remains to be determined how in our model NOX2 senses reductions in O-GlcNAc levels to activate the downstream pathway leading to p38 phosphorylation.

In addition to the canonical MKK3/6 leading to p38 activation during OGT inhibition, we identified a second mechanism of p38 activation that involves Tab1. Tab1 serves as a scaffold protein that recruits p38 to induce its autoactivation (75). This mode of activation has been described in many cell types including cardiac myocytes where it modulates p38 activity (90). Here, we found that the knockdown of Tab1 reduced OSMI-1–induced phosphorylation of p38. Notably, Tab1 is a known substrate of OGT (76, 91) and in agreement, we find here that a subset of Tab1 is O-GlcNAcylated in cardiac myocytes. Previously, it has been reported that noncanonical p38 activation is under negative regulation of Hsp90/Cdc37 in cardiac myocytes (74). Consistently, we found that inhibiting this complex with geldanamycin increases p38’s phosphorylation, which is additive to that induced by OSMI-1, suggesting that the activating effect of OGT inhibition occurs downstream of the release of p38 from its inhibitory complex. Interestingly, we find that like Tab1, Hsp90 also appears to be O-GlcNAcylated in cardiomyocytes. In a potential scenario, OSMI-1 inhibition of OGT could lead to a net loss of O-GlcNAc from Hsp90 and Tab1, which would then facilitate the release from the former and increased interaction with the latter leading to activation of p38. It is noteworthy that the minimum region of Tab1 required for p38 autoactivation, the 46-mer 371 to 416 (92), encompasses a well-known O-GlcNAcylation site at S395 (76). However, whether O-GlcNAcylation on S395 is critical for p38 activation during stress conditions has yet to be determined and a knock-in mouse with a S395A substitution exhibits a normal baseline phenotype (93).

Results from OGT assays suggest that p38 can be O-GlcNAcylated *in vitro* (94). In agreement, using the metabolic labeling approach, coupled with pull-down and Western blotting, we found that p38 was O-GlcNAcylated in a manner sensitive to OSMI-1. There is currently a paucity of known O-GlcNAcylation sites on p38 and consequently, it is difficult to rationalize how the presence or absence of O-GlcNAc on such sites might impact p38 activity. In the simplest scenario, it could be that O-GlcNAcylation on T180 within p38’s activation loop directly hinders its phosphorylation either by MKK3/6 or during autoactivation. Furthermore, it was found that a highly conserved threonine within the activation loop, T185, is essential for the Tab1-mediated autoactivation of p38 (95). Whether O-GlcNAcylation on the aforementioned, or other unknown sites, modulates the activation of p38 warrants further investigation. Finally, it is worth mentioning that enhancing O-GlcNAcylation by glucosamine perfusion blunts the phosphorylation of p38 during cardiac ischemia but increases it during reperfusion, further underscoring a tight but complex relationship between O-GlcNAcylation and p38 phosphorylation in the intact heart (11).

The activity of Erk1/2 in cardiomyocytes and hearts is generally associated with physiological responses while that of p38 is associated with pathophysiological outcomes (96). In the context of OGT inhibition for 24 h, it could be that the simultaneous disruption of Erk1/2 and p38 signaling contributes to the impaired hypertrophic response to PE. After treating cells with OSMI-1 for 24 h, we found decreased phosphorylation of Erk1/2 and increased phosphorylation of p38. Erk1/2 signaling induces prohypertrophic protein synthesis (97), and inhibiting Erk1/2 blunts the adaptive cardiac hypertrophy (98). Consistently, in our experimental model, we find that direct Erk1/2 inhibition prevents PE-induced hypertrophic growth, and similarly, reduced Erk1/2 phosphorylation in the context of OGT inhibition correlates with a blunted PE-induced myocyte hypertrophy. Along these lines, the PE-induced expression of *cTnT*, a gene encoding for a key component of myofilaments, is downregulated by Erk1/2 inhibition, and this response is also recapitulated during OGT inhibition. Compared to that of Erk1/2, the role of p38 in the context of PE-induced hypertrophy is limited (99) and consistently, the p38 inhibitor alone did not significantly block the PE-induced hypertrophic growth. Overactive p38 signaling in the heart induces a fetal gene expression signature, including increased Anp expression (100). While we found that the induction of *Anp* downstream of PE stimulation is largely driven by Erk1/2, it is still possible that overactive p38 compensates for the decreased Erk1/2 signaling and contributes to the uniformly high levels of *Anp* expression in the context of PE stimulation and OGT inhibition. Consistently, both Erk1/2 and p38 are reported to have a convergent role in the regulation of promoters controlling the expression of natriuretic peptides (101).

Cardiomyocyte-specific deletion of OGT leads to early postnatal lethality due to developmental defects (102, 103), highlighting OGT’s role in cardiomyocyte growth and maturation. In agreement, our work with primary neonatal cardiomyocytes found that OGT inhibition blunts hypertrophic growth induced by PE. Consistent with an impaired growth response, OSMI-1 did not reverse the PE-induced expression of pathologic markers *Anp* and *Bnp* and it downregulated PE-induced *cTnT* expression. Together with the knockout results, our findings underscore the important role of OGT in developmental cardiomyocyte hypertrophy. In the adult heart, O-GlcNAcylation is upregulated during the early response to pressure overload, indicating its importance in adaptive hypertrophy (104, 105). Consistently, hearts with adult-onset OGT deficiency exhibit systolic dysfunction after pressure overload, although this is not accompanied by significant changes in cardiomyocyte size and overall cardiac mass (14, 15). Collectively, it appears that OGT activity promotes cardiomyocyte growth in postnatally developing hearts and maintains cardiomyocyte function/contractility during early pressure-overload hypertrophy in adult hearts. Interestingly, a balanced cardiomyocyte OGT activity needs to be kept, because mice with cardiac overexpression of OGT throughout embryonic and postnatal development exhibit cardiomyopathy by early adulthood (21).

The underlying mechanisms leading to cardiac dysfunction in hearts with altered OGT expression either at baseline or after challenge with pressure overload are incompletely understood and no immediate connections have been made for the implications of MAPK signaling. However, one study found that the transcription factor Gata4 was strongly downregulated in OGT-deficient hearts challenged with pressure overload (14). Because Gata4 is a downstream target of Erk1/2 signaling, its downregulation in OGT-deficient heart might indicate a dysregulation of Erk1/2 signaling. Erk1/2 signals through Gata4 to mediate adaptive hypertrophy in cardiac myocytes (106, 107) and consistently, our findings here show that OGT inhibition perturbs Erk1/2 phosphorylation concomitantly with blunted hypertrophy. Interestingly, we find that Gata4 is O-GlcNAcylated in agreement with previous findings in the heart (108). While O-GlcNAcylation appears to promote the transcriptional activity of Gata4 (108), it is unknown whether reduced O-GlcNAcylation can lead to its downregulation. Ultimately, it would be interesting in future studies to address to what extent the gain of Gata4 could reverse any of the phenotypes of OGT inhibition observed here.

In this study, we have used inhibitors extensively, which offers the advantage of acute target manipulation but also has disadvantages such as off-target effects or unexpected activities (109). Compound SB202190 (an ATP-binding pocket interactor) exhibits high selectivity for p38α and p38β (61), however, it was found to interact with 12 other targets from a panel of 119 protein kinases (110). Inhibitor SCH772984 (binding to an allosteric pocket) exhibits high selectivity for Erk1 and Erk2 against a panel of 456 kinases but can also interact with 12 other kinases with greater than 90% binding affinity (111). Inhibitor GS-444217 (interacting with the ATP-binding pocket and thus serving as a competitive inhibitor) exhibits high selectivity for Ask1 with only two other potentially interacting kinases across a panel of 442 kinases (112). Inhibitor takinib (ATP-binding domain interactor) exhibits high selectivity for Tak1, targeting only five other kinases across a panel of 140 kinases albeit with substantially higher IC_50_s (113). On the other hand, compound URMC-099 (a likely ATP-binding pocket interactor) exhibited only moderate selectivity for Mlk3 as it interacted with 111 other kinases across a panel of 442 (114). Finally, geldanamycin, an Hsp90-binding macrocyclic, was found to affect the expression of 288 kinases (115). Taken together, most of these compounds are highly selective for their intended kinases, although their potential targeting of other proteins (kinases or not) necessitates the use of caution when interpreting their effects on p38 and Erk1/2 signaling pathways examined here.

TMG was tested against human lysosomal β-hexosaminidase and five other glycoside hydrolases and was found to be highly selective for OGA with a K_i_ value of 21 nM and an IC_50_ in the nanomolar range (70). Consistently, in our experiments, we found that while significantly increasing O-GlcNAcylation, TMG did not impact the abundance of cell surface N- or O-linked glycans. OSMI-1 was developed from a quinoline-6-sulfonamide scaffold and was found to target OGT with an IC_50_ of 2.7 μM and dose dependently decreased O-GlcNAcylation in cells (69). Due to the lack of glycosyltransferase panels (akin to kinase panels), screening for off-target binding of OSMI-1 to other glycosyltransferases was not possible. Nevertheless, screening with lectin blots did not identify a gross impact of OSMI-1 on the abundance of various N- and O-linked glycans (69). Similarly, lectin blots with cardiomyocyte extracts performed here did not reveal a significant impact of OSMI-1 on cell surface N- and O-linked glycans. It should be noted that a close structural analog of OSMI-1, PG34, had a sizable negative effect on cell viability after 24 h of exposure despite being a weak OGT inhibitor (IC_50_ = 68 μM), indicating that OSMI-1 might potentially have off-target effects impacting cell viability (69). In light of this caveat and while caution should be used in interpreting the results, it should be noted that the majority of the experiments done here involved acute use of OSMI-1 for up to 6 h at an intermediate concentration (25 μM). Furthermore, although not designed to test cell viability *per se*, our experiments assessing myocyte hypertrophy found that baseline (non-PE–treated) cells exposed to OSMI-1 for 24 h did not have lower nuclear counts.

In summary, we have uncovered a functional relationship between O-GlcNAcylation and MAPK signaling in cardiomyocytes and have identified several potential entry points for regulation. While we provide evidence for O-GlcNAcylation on a number of proteins within the MAPK signaling pathway (*e.g.*, MEK1/2, Erk1/2, Tab1, p38, Hsp27, and others), it remains an open question whether O-GlcNAcylation on this handful of proteins or on other substrates constitutes the key driving mechanism behind the observed impairment of PE-induced growth of cardiomyocytes. Therefore, further investigation is warranted to identify the most relevant substrate proteins and sites. The work presented here paves the way for such target-focused studies that can ultimately provide new approaches for intervening in the MAPK pathway in cardiac pathophysiology.

## Experimental procedures

### Materials

A detailed list of key reagents including their identifiers, vendors, and catalog numbers is provided in Table S1. Additional information on other reagents can also be found in the description of methods in the text below. The primer sequences used in quantitative real-time PCR are shown in Table S2. Procedures involving the use of animals were approved by the Institutional Animal Care and Use Committees (IACUC) at the Johns Hopkins School of Medicine.

### Primary neonatal myocyte culture and treatments

NRVMs were isolated from P0-P1 rats of either sex using enzymatic dissociation, according to a standard methodology as previously described (116, 117). Following a preplating step, to remove fibroblasts and other nonmyocytes, the cardiomyocytes (suspensions at 0.5 × 10^6^ cells/ml in Dulbecco’s modified Eagle’s medium supplemented with 10% FBS) were seeded for experiments on culture plates precoated with 0.1% bovine gelatin (Cat. No. G9391, Sigma) and incubated at 37 °C, 5% CO_2_ for 24 h. Typical seeding densities were 1.0 × 10^6^ cells per well in a 6-well plate for Western blot experiments or 1.25 × 10^5^ cells per chamber in ibidi μ-Slide (Cat. No. 80426) for confocal microscopy. On the next day, the cells were washed twice with prewarmed PBS and the medium was switched to 0% FBS Dulbecco’s modified Eagle’s medium (Cat. No. 10-013-CV, Corning, 4.5 g/l glucose) supplemented with insulin transferrin selenium (Cat. No. 51500056, Gibco). Following 24 h of serum starvation, the cells were stimulated with the indicated agonists and/or inhibitors for the specified durations for each experiment (see also main text and/or figure legends). In experiments for gene knockdown, cells were transfected with a lipofectamine/siRNA mix at the time of seeding. Briefly, dicer-substrate short interfering RNA was complexed with Lipofectamine RNAiMax (Cat. No. 13778075, Thermo Fisher Scientific) at room temperature for 15 min according to manufacturer’s specifications and the transfection mix was added to the cell suspension at a ratio of 40 pmol siRNA/1 × 10^6^ cells.

### Protein extraction and immunoblotting

Cells were washed with ice-cold PBS (3×) and lysed in RIPA buffer (contains 150 mM NaCl, 1.0% IGEPAL CA-630, 0.5% sodium deoxycholate, 0.1% SDS, 50 mM Tris, pH 8.0, Cat. No. R0278, Sigma) supplemented with 2 μM TMG, 1 mM PMSF, and 1× protease and phosphatase inhibitors (cOmplete, mini, EDTA-free, and PhosSTOP respectively, Millipore, Sigma). Lysates were sonicated and protein concentration was quantified with the bicinchoninic acid assay (Cat. No. 23225, Pierce). For Western blot, protein samples were combined with 50 mM DTT and 1× LDS sample buffer (NP0008, NuPAGE, Thermo Fisher Scientific), and 10 μg total protein per lane was resolved by electrophoresis, typically run in NuPAGE Bis-Tris 4 to 12% gradient gels (Cat. No. WG1403, Thermo Fisher Scientific). Proteins were transferred onto nitrocellulose membranes (iBlot2 transfer stacks, Cat. No. IB23001, Thermo Fisher Scientific). The membranes were blocked with 5% bovine serum albumin (BSA) in TBS for 1 h at room temperature and then incubated with primary antibodies, typically at a 1000:1 dilution in 5% BSA in 0.1% Tween20-TBS, overnight at 4 °C (see Table S2 for a list of primary antibodies used). For detection, we used the LI-COR Odyssey system and appropriate host-specific secondary antibodies (*e.g.*, IR Dye 800w goat anti-rabbit, Cat. No. 926-32211, LI-COR) typically at a 5000:1 dilution in 5% BSA in 0.1% Tween20-TBS. For the detection of biotinylated targets, we used IRDye800w Streptavidin (Cat. No. 926-32230, 5000:1 in % BSA in 0.1% Tween20-TBS). Quantification of band intensities was performed with Image Studio Lite (LI-COR). Normalization for protein loading was performed based on intensities obtained by staining the membranes with REVERT 700 Total Protein Stain (Cat. No. 926-11010, LI-COR).

For the detection of glycans with lectin blotting, we used lectin kit 1 (Cat. No. BK-1000, Vector Laboratories) that includes biotinylated conjugates of wheat germ agglutinin, ConA, peanut agglutinin, and *D. biflorus* agglutinin. For the lectiblots, proteins were run on NuPAGE Bis-Tris 4 to 12% gradient gels and then were transferred using the TransBlot Turbo system (Bio-Rad) onto nitrocellulose membranes (kit Cat. No. 1704271, Bio-Rad). Following blocking with 5% BSA in TBS, membranes were incubated with 5000:1 diluted lectins (final concentration of lectin 0.4 μg/ml) overnight at 4 °C. Following extensive washes with TBS-T, the bound biotinylated lectins were detected with IRDye800w Streptavidin. To control for the reactivity of streptavidin with endogenously biotinylated proteins, some experiments were done as negative controls where no lectin was included in the overnight step. These experiments yielded reactive bands at ∼75 and also at 125 and 250 kDa.

### Metabolic labeling with unnatural sugar analogs, alkyne-azide click reaction, and enrichment of putative O-GlcNAcylated proteins

The procedure was developed based on protocols described previously (65, 66, 67). Briefly, for the metabolic labeling of glycans with clickable handles, NRVM or HEK293 cells were incubated in growth medium supplemented with 200 μM Ac4GalNAz or 200 μM Ac4GalNAlk for 24 h. Following metabolic labeling, the cells were washed in ice-cold PBS (3×), lysed in a buffer containing 1% SDS, 2 μM TMG, protease and phosphatase inhibitors, 50 mM Tris HCl pH 8.0, and sonicated as described above. In some experiments, after 18 h of treatment with the metabolic chemical reporter, the cells were treated with vehicle, TMG (200 nM), or OSMI-1 (25 μM) for an additional 6 h in the presence of the metabolic reporter. For the labeling of glycosylated proteins with CalFluor 647 azide, the copper-catalyzed alkyne-azide reaction contained 20 μg protein suspension in 0.6% SDS, 2 mM sodium ascorbate, 100 μM THPTA, 1 mM CuSO_4_.5H_2_O, and either 20 μM or 50 μM CalFluor 647 azide (Cat. No. 1372, Click Chemistry Tools). As a negative control for the click reaction, a set of reactions was set up as above in the presence of 50 μM CalFluor 647 azide, omitting the catalyst Cu/THPTA. The ‘click’ reaction took place for 1 h at room temperature in the dark and was quenched with 5 mM EDTA. Then, samples were mixed with 1× LDS sample buffer and 50 mM DTT and were resolved on a 4 to 12% Bis-Tris gradient gel which was subsequently scanned on the LI-COR Odyssey system using the 700 channel (685 nm infrared laser).

In other experiments, lysates from Ac4GalNAz- or Ac4GalNAlk-treated cells were used in copper-catalyzed alkyne-azide reactions containing 500 μg protein suspension in 0.6% SDS, 2 mM sodium ascorbate, 100 μM THPTA, 1 mM CuSO4.5H2O, and 40 μM biotin azide plus (Cat. No. 1488, Click Chemistry Tools). Following ‘click’ reaction and quenching, the reaction mix was cleared by methanol/chloroform precipitation as described above and proteins resuspended in 1.0% SDS. A fraction of this suspension was taken as ‘input’. Next, high-capacity streptavidin-agarose slurry (Cat. No. 20357, Pierce) corresponding to 100 μl settled resin was placed into spin columns (Cat. No. 89868, Pierce) and washed twice in water and then twice in 50 mM Tris HCl, pH 8.0. Washes were done by spinning at 1500*g* for 1 min and discarding the flow-through. The protein suspension was then diluted 5-fold in 50 mM Tris HCl, pH 8.0 (0.2% SDS final concentration), added to the washed streptavidin resin bed, and allowed to bind for 2 h at room temperature with end-over-end rotation. Afterward, the suspension was spun to obtain the ‘unbound’ fraction, while the agarose beads were washed 5× with IP wash buffer (1% NP-40, 150 mM NaCl, 50 mM Hepes, pH 7.9). To elute biotinylated proteins from the beads, we used a solution containing 80% acetonitrile, 0.1% formic acid, and 0.2% TFA and heating at 65 °C for 5 min (118). The released biotinylated proteins were separated from the beads by spinning at 1500*g* for 1 min and the eluted fractions were dried down by speed-vac centrifugation. The dried protein pellets were resuspended in 1× LDS/50 mM DTT sample buffer and run next to ‘input’ and ‘unbound’ fractions by gel electrophoresis and Western blot. Detection of eluted proteins was performed with IRDye800w streptavidin for total biotinylated proteins or with appropriate antibodies for specific intracellular proteins of interest.

### RNA isolation, reverse transcription, and gene expression analysis by quantitative real-time PCR

Total RNA was extracted from NRVMs seeded at 1.0 × 10^6^ cells/well after appropriate treatments as indicated in the main text (*e.g.*, 24-h PE treatment ± TMG/OSMI-1 treatment). The treated cells were washed with ice-cold PBS (3×) and RNA was extracted with the RNeasy kit (Cat. No. 74704, Qiagen), including a proteinase K digestion step to remove abundant sarcomeric proteins. In-column DNaseI treatment (Cat. No. 79254, Qiagen) was used to digest away genomic DNA. For reverse transcription, we used a QuantiTect reverse transcription kit (Cat. No. 205311, Qiagen) and an input of 850 ng of total RNA. To control for potentially residual genomic DNA carry-over, reactions without reverse-transcriptase were set up in parallel. Real-time quantitative PCR was performed with the Power SYBR Green master mix (Cat. No. 4367659, Thermo Fisher Scientific) in the presence of 500 nM forward and reverse primers (for gene-specific sequences see Table S2). A total of 45 cycles of amplification were done (15 s at 95 °C for melting followed by 30 s at 60 °C for annealing and elongation) using the CFX384 Touch system (Bio-Rad). Each sample was run in technical triplicates. Gene expression was quantified with the ΔC_t_ method using Gapdh and 36B4 as housekeeping genes.

### Cell processing for F-actin staining, imaging, and quantitation of NRVM hypertrophy

Following seeding and treatments in 4-chamber imaging slides, NRVMs were washed with 37 °C prewarmed PBS (3×) and then fixed with prewarmed 4% paraformaldehyde for 15 min. The fixative was then washed off with PBS and cells were permeabilized with 0.1% Triton X-100 for 5 min at room temperature. Permeabilization was followed by PBS washes and finally blocking overnight at 4 °C with 1% BSA in 0.1% tween-20 PBS. To stain F-actin, we exposed cells to 5 units/ml phalloidin-Alexa 594 (Cat. No. A12381, Thermo Fisher Scientific) diluted in 1% BSA, 0.1% Tween-20, PBS for 1 h at room temperature. An additional 15-min staining step was done with 5000-fold diluted Hoechst 33342 (Cat. No. H3570, Thermo Fisher Scientific) to stain nuclei, and then cells were washed with PBS. Imaging was carried out with the FV3000RS confocal microscope (Olympus) using the 10× objective (UPLSAPO10X2, numeric aperture 0.40, working distance 3.1 mm) and 405 nm and 561 nm lasers to excite Hoechst and Alexa 594, respectively. Laser power and gain were kept constant across imaging sessions and four fields of view were captured per chamber.

For data analysis, .oir images were uploaded into a Fiji-run custom-made macro to convert composite images into individual.tiff single-color channels (blue and red). The images were then uploaded into Cell Profiler (latest version 4.2.1., https://cellprofiler.org/). The Cell Profiler pipeline extracts nuclei from the blue channel and uses those as primary objects. Next, using nuclei as primary objects, the pipeline identifies the cells from the red channel (phalloidin) using the ‘Propagation’ method. Next, the cytoplasm is identified by subtracting the nuclear area from the cell area. The main output parameters of the pipeline utilized for quantification of the F-actin signal were the ‘Integrated Cytoplasmic Intensity’ measured as the intensity of the phalloidin signal in the cytoplasmic area. The mean integrated cytoplasmic intensity across all cells within a given field of view was used as a single data point. Additionally, the ‘Nuclear Counts’ measured as the total number of nuclei in a given field of view were used as a secondary parameter of overall cell viability/representation in a given field of view to confirm that sampling was performed from areas with comparable cell density.

### Statistical analysis

Data were handled and plotted using Microsoft Excel and GraphPad Prism. Values in the graphs are reported as means ± standard error. The numbers of biological replicates are shown inscribed in their respective bars. Data were examined for normality of distribution using the Shapiro-Wilk test and then visually inspected (*e.g.*, residual plot and Q-Q plot) for any potential outliers. If necessary, one or more outliers were identified using the ROUT test (Q = 1%). To identify statistically significant differences between two groups, we performed unpaired student’s *t* test. When comparing three or more groups, we used one-way or two-way ANOVA depending on the number of factors (*e.g.*, for comparing the effect across three or more drugs, we used one-way, whereas for comparing groups resulting from a combination of drugs and gene manipulation, we used two-way). To identify statistically significant differences, the data were assessed for normality of distribution. If the majority of groups within an experiment had normally distributed data, we used the Tukey *post hoc* test. If the distribution of the data in the majority of groups was not normal, we used the Dunnet *post hoc* test. In all cases, the α level for statistical significance was 0.05. *p* values less than 0.05, 0.01, 0.001, and 0.0001 are symbolized as ∗, ∗∗, ∗∗∗, and ∗∗∗∗ respectively.

## Data availability

All data presented are included in the article and can be available upon request from Kyriakos N. Papanicolaou, Johns Hopkins University School of Medicine, kyriakos@jhu.edu.

## Supporting information

The article contains supporting information.

## Conflict of interest

The authors declare no competing interests.
