# Supplementary material for: Inhibiting O-GlcNAcylation impacts p38 and Erk1/2 signaling and perturbs cardiomyocyte hypertrophy
Source: J Biol Chem. 2023 Jan 13;299(3):102907. doi: 10.1016/j.jbc.2023.102907 (PMC9988579; doi:10.1016/j.jbc.2023.102907)
Supplement: Supporting Figures S1–S4 and Tables S1 and S2 [file mmc1.pdf]

Supporting information

Inhibiting O-GlcNAcylation impacts p38 and Erk1/2 signaling and perturbs cardiomyocyte hypertrophy

Kyriakos N. Papanicolaou et al.

Contents

Supporting Figures S1-S4
Supporting Tables S1-S2

Supporting Figure S1

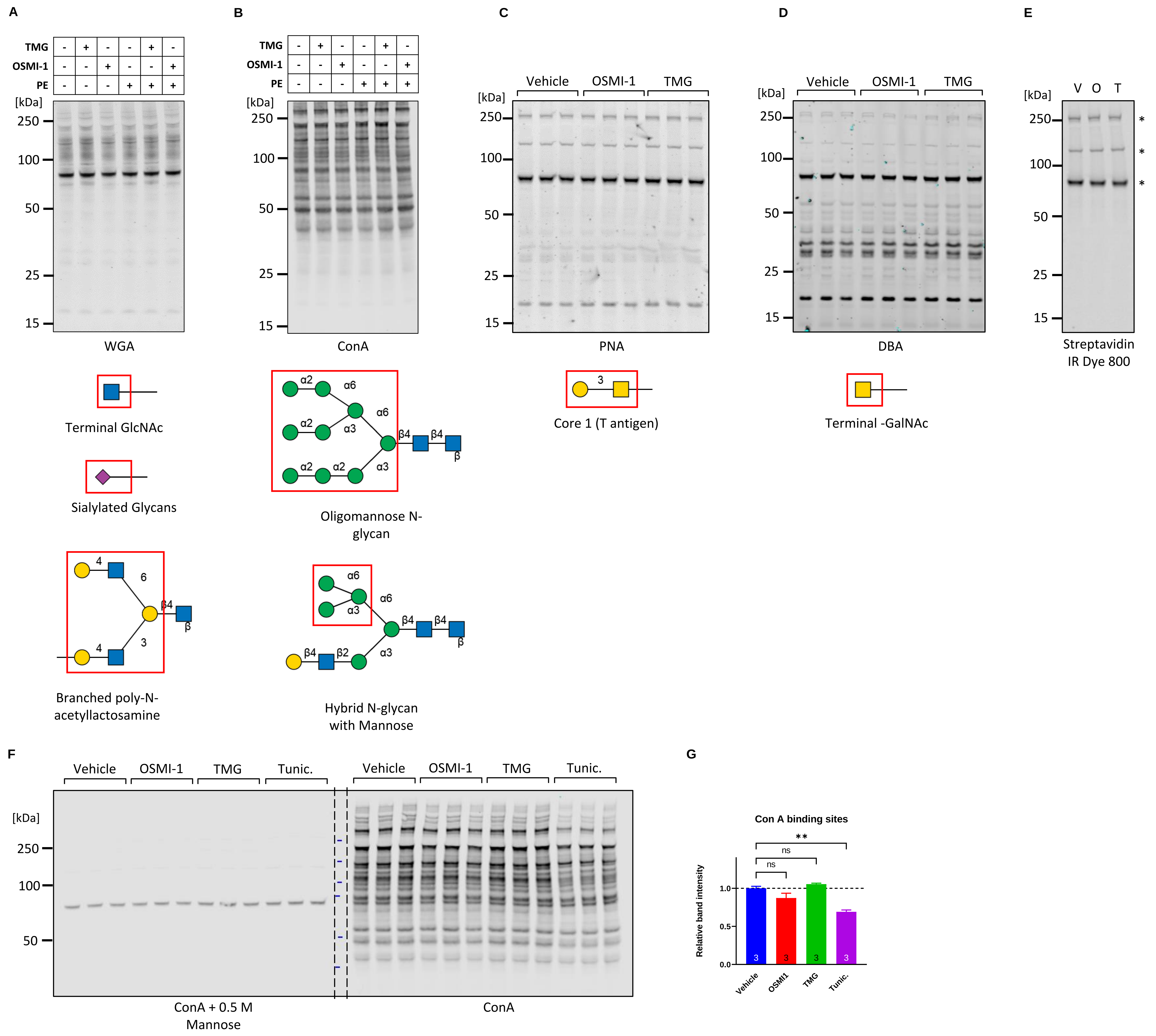

Supporting Figure S1. TMG and OSMI-1 increase and decrease protein O-GlcNAcylation without impacting the overall abundance of other glycans

(**A-B**) NRVMs were exposed to 200 nM Thiamet-G (TMG), or 25 µM OSMI-1 and were harvested 6 hours later for lectin blot analysis. Wheat germ agglutinin (WGA, 0.4 µg/ml) was used to detect changes in glycans bearing GlcNAc or sialic acid (see also glycan structures depicted below blots where the binding epitope of each lectin is shown in red squares). Concanavalin A (ConA, 0.4 µg/ml) was used to detect changes in N-linked high- mannose structures. (**C-D**) Samples from the indicated treatment groups were analyzed with lectin blots using peanut agglutinin (PNA) or *Dolichus biflorus* agglutinin (DBA) to detect differences in the abundance of glycans bearing ‘core 1’ or terminal GalNAc respectively (final concentration of each lectin 0.4 µg/ml). (**D**) Control blot without any lectin indicating the presence of endogenous biotinylated proteins that cross-react with IRDye800w Streptavidin. These proteins (migrating at ~75, 125 and 250 kDa) are not necessarily glycosylated and are denoted with asterisks. (**F-G**) Samples from the indicated treatment groups were incubated with ConA that was competed with 500 mM free mannose (left half) or without competition (right half). Tunicamycin was used at 4 µg/ml for 6 hrs. Comparisons were done with one-way ANOVA and Tukey *post-hoc* test. ** P <0.01, ns; not significant.

Supporting Figure S2

A

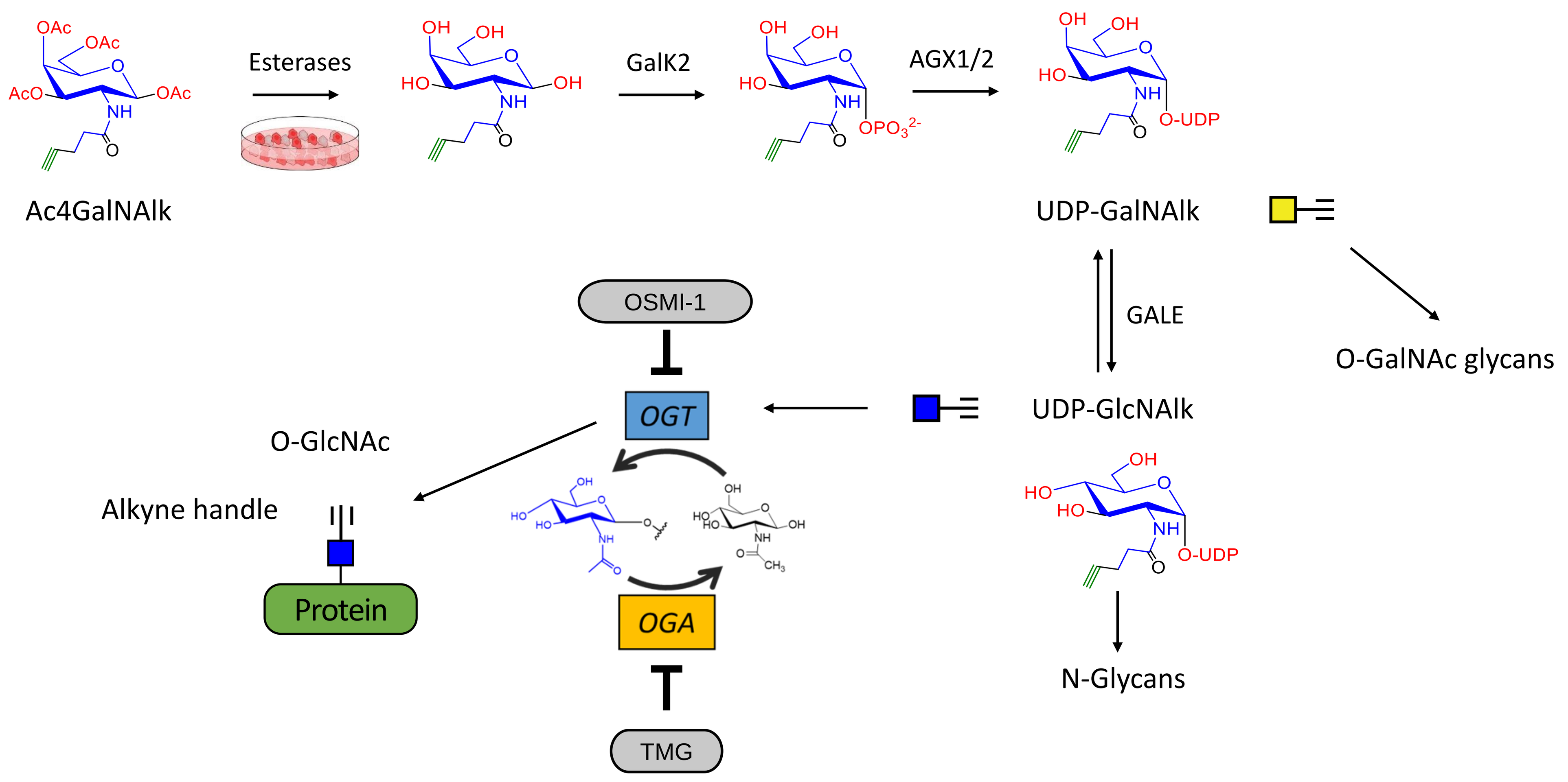

B

**Ac4GalNaz**	+	+	+	-	-	-	+	+	+	-	-	-	+	+	+	-	-	-
**Ac4GalNalk**	-	-	-	+	+	+	-	-	-	+	+	+	-	-	-	+	+	+
**Vehicle**	+	-	-	+	-	-	+	-	-	+	-	-	+	-	-	+	-	-
**TMG**	-	+	-	-	+	-	-	+	-	-	+	-	-	+	-	-	+	-
**OSMI-1**	-	-	+	-	-	+	-	-	+	-	-	+	-	-	+	-	-	+
**Fraction**	**5% input**						**5% unbound**						**eluate**					

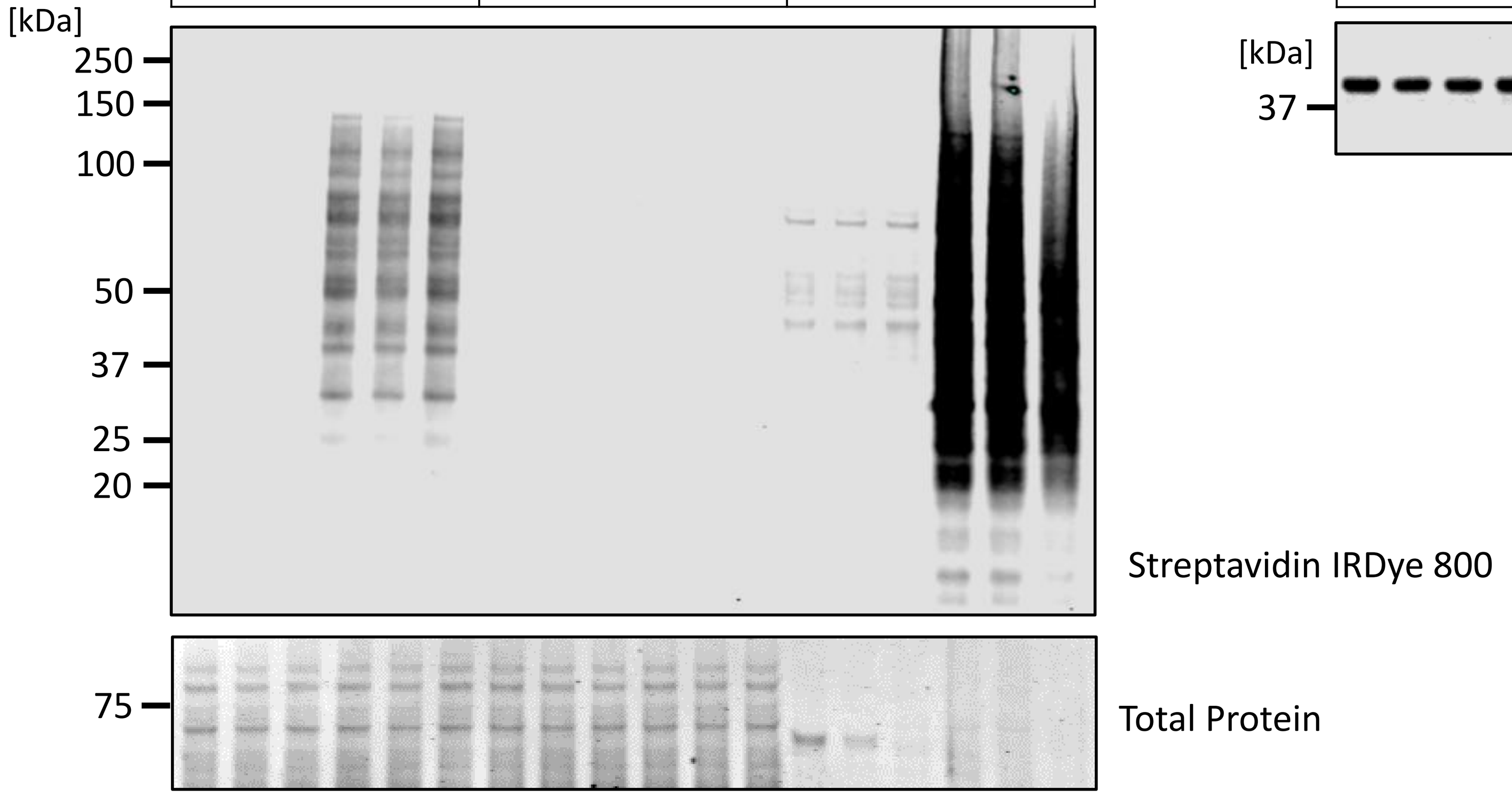

C

**Ac4GalNaz**	+	+	+	-	-	-	+	+	+	-	-	-
**Ac4GalNalk**	-	-	-	+	+	+	-	-	-	+	+	+
**Vehicle**	+	-	-	+	-	-	+	-	-	+	-	-
**TMG**	-	+	-	-	+	-	-	+	-	-	+	-
**OSMI-1**	-	-	+	-	-	+	-	-	+	-	-	+
**Fraction**	**5% input**						**eluate**					

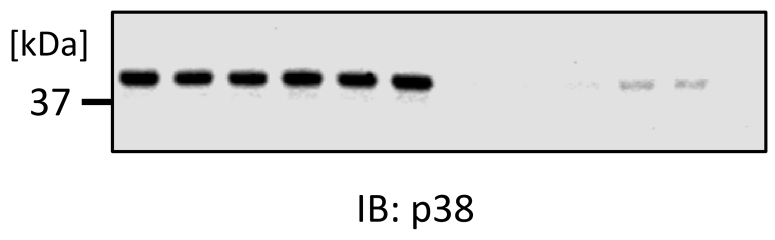

Supporting Figure S2. Metabolic labeling and enrichment of glycosylated p38 is sensitive to OSMI-1.

(**A**) Schematic of the metabolic pathway that converts the unnatural sugar Ac4GalNAlk into UDP-GalNAlk and UDP-GlcNAlk that can then be incorporated into diverse glycans of the cell. Galk2; N-acetylgalactosamine kinase, AGX1/2; UDP-N-acetylhexosamine pyrophosphorylase; GALE; UDP-Galactose epimerase. The schematic shows how UDP-GlNAlk can be used up by OGT to label O-GlcNAcylated proteins (**B**) HEK293 cells were metabolically labelled with Ac4GalNAz or Ac4GalNAlk (200 µM) for 24 hours followed by an additional 6-hour exposure to TMG (200 nM) or OSMI-1 (25 µM). Extracted proteins (100 µg protein) were reacted with biotin azide plus in 'click' reactions. Biotinylated proteins were enriched by streptavidin pull-down and fractions from all treatment groups were blotted onto nitrocellulose membranes and probed by streptavidin IR dye 800 to assess enrichment. (**C**) The glycosylation of p38 and its sensitivity to OSMI-1 was assessed by immunoblotting in samples resulting from biotin-streptavidin pull downs in the different treatment groups.

Supporting Figure S3

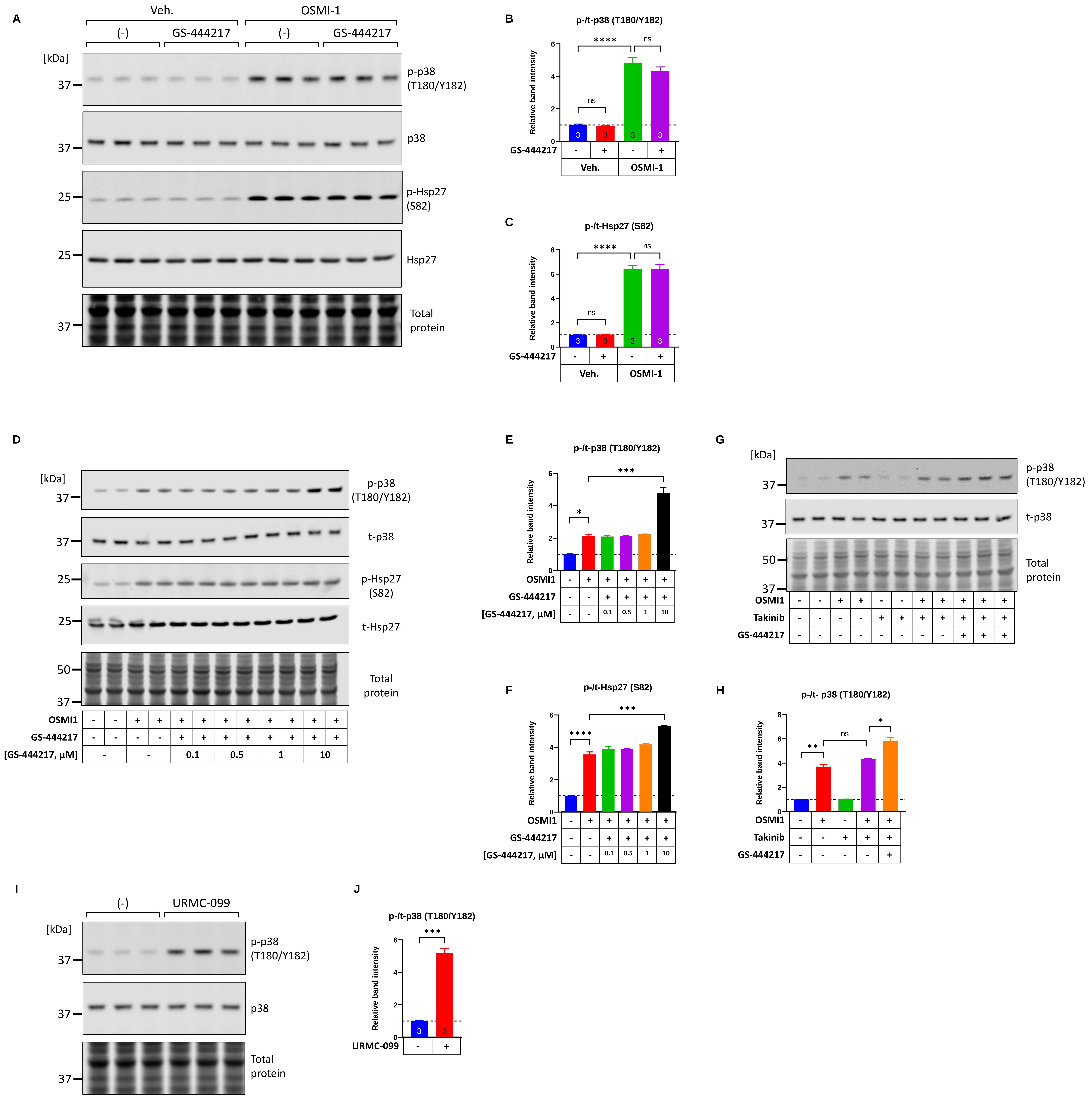

Supporting Figure S3. Variable effects of inhibitors GS-444217, takinib and URMC-099 on OSMI-1-induced p38 phosphorylation. (**A-C**) NRVMs were treated with the Ask1 inhibitor GS-444217 (1 µM) alone or in combination with OSMI-1 (25 µM) for 6 hours after which protein extracts were analyzed for p38 and Hsp27 phosphorylation. (**D-F**) NRVMs were treated with OSMI-1 (25 µM), alone or in combination with the Ask1 inhibitor GS-444217 (increasing concentrations from 0.1 to 10 µM) for 6 hours after which protein extracts were analyzed for p38 and Hsp27 phosphorylation. (**G-H**) NRVMs were treated with OSMI-1 (25 µM), takinib (10 µM) with or without the addition of GS-444217 (10 µM) and cell extracts were analyzed after 6 hours of treatment for p38 phosphorylation. (**I-J**) NRVMs were treated with the Mlk3 inhibitor URMC-099 (10 µM) and analysed 6 hours after treatment for the phosphorylation of p38. Statistical differences were assessed by one-way ANOVA and Tukey *post-hoc* test. * *P* <0.05, ** *P* <0.01, *** *P* <0.001, **** *P* <0.0001, ns; not significantly different.

Supporting Figure S4

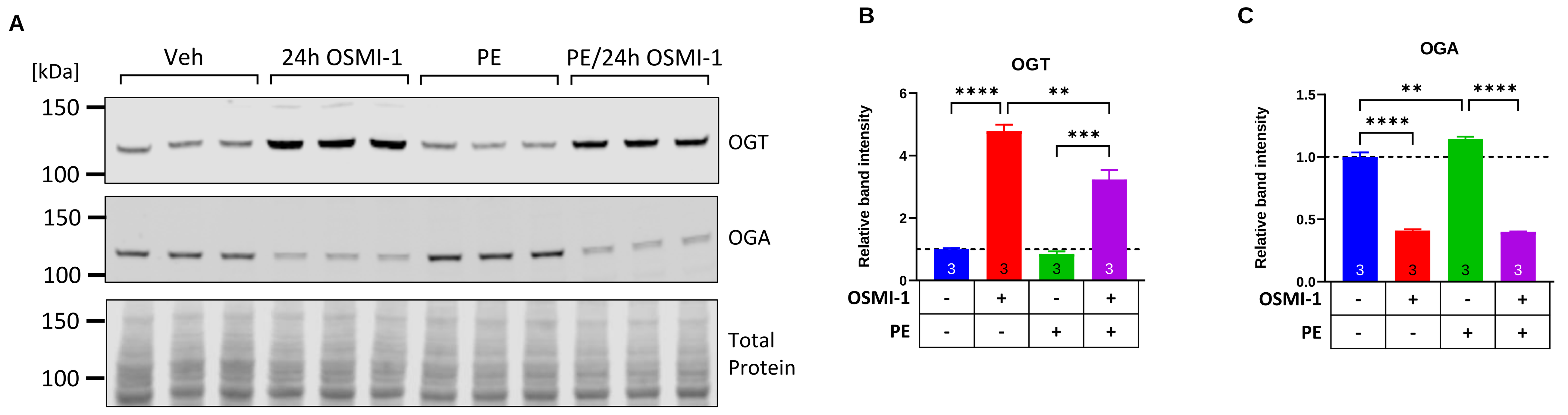

Supporting Figure S4. Sustained OGT inhibition with OSMI-1 increases protein levels of OGT and decreases the protein levels of OGA

(**A-C**) Western blot and quantitation of OGT and OGA protein abundance after exposure to OSMI-1 (25 µM,24 hours) with or without PE stimulation (5 µM, 30 min.) . Comparisons across treatment groups were done with one-way ANOVA and Tukey post-hoc test. ** P <0.01, *** P <0.001, **** P <0.0001.

Supporting Table S1. Reagent list

REAGENT	SOURCE	IDENTIFIER
**Antibodies**		
**Mouse monoclonal anti-O-GlcNAc**	Zachara Lab	RL2
**Rabbit monoclonal anti- MGEA5/OGA**	Abcam	Cat# ab124807, RRID:AB_10971848
**Rabbit monoclonal anti- OGT (D1D8Q)**	Cell Signaling Technology	Cat# 24083, RRID:AB_2716710
**Rabbit monoclonal anti-phospho-p38 MAPK (Thr180/Tyr182) (D3F9)**	Cell Signaling Technology	Cat# 4511, RRID:AB_2139682
**Rabbit polyclonal anti-p38 MAPK Antibody,**	Cell Signaling Technology	Cat# 9212, RRID:AB_330713
**Rabbit polyclonal anti- Phospho-HSP27 (Ser82) Antibody**	Cell Signaling Technology	Cat# 2406, RRID:AB_2120485
**Rabbit polyclonal anti- HSP27 Antibody (Rodent Preferred)**	Cell Signaling Technology	Cat# 2442, RRID:AB_2233273
**Rabbit monoclonal anti- Phospho-CREB (Ser133) (D1G6)**	Cell Signaling Technology	Cat# 4276, RRID:AB_10544696
**Rabbit monoclonal anti- CREB (D76D11)**	Cell Signaling Technology	Cat# 4820, RRID:AB_1903940
**Rabbit monoclonal anti- Phospho-p44/42 MAPK (Erk1/2) (Thr202/Tyr204)**	Cell Signaling Technology	Cat# 4370, RRID:AB_2315112
**Rabbit monoclonal anti- p44/42 MAPK (Erk1/2) (137F5)**	Cell Signaling Technology	Cat# 4695, RRID:AB_390779
**Rabbit monoclonal anti- MEK1/2**	Cell Signaling Technology	Cat# 9122, RRID:AB_823567
**Rabbit polyclonal anti-Gata4**	Proteintech	Cat# 19530-1-AP, RRID:AB_10642003
**Rabbit monoclonal anti-MAPK / CDK Substrates (PXSP or SPXR/K)**	Cell Signaling Technology	Cat# 2325, RRID:AB_331820
**Rabbit polyclonal anti-Tab1**	Proteintech	Cat# 27566-1-AP, RRID:AB_2880910
**Rabbit polyclonal anti- Phospho-Tab1 (T431)**	Millipore	Cat# 06-1334, RRID: AB_10807031
**Rabbit polyclonal anti-Hsp90AB1**	Sigma-Aldrich	Cat# SAB4300541, RRID: AB_10629523
**Lectin Kit I, biotinylated**	Vector Laboratories	Cat# BK-1000, RRID:n/a
**Streptavidin IR Dye800w**	LiCor	Cat# 926-32230 RRID: n/a

**Chemicals**		
**OSMI-1**	Sigma-Aldrich	SML1621; CAS No.: 1681056-61-0
**Thiamet-G**	Tocris	Cat# 4390; CAS No.: 1009816-48-1
**SB202190**	MedChemExpress	HY-10295; CAS No.: 152121-30-7
**SCH772984**	MedChemExpress	HY-50846; CAS No.: 942183-80-4
**(R)-(−)-Phenylephrine hydrochloride**	Sigma-Aldrich	P6126; CAS No.: 61-76-7
**Tunicamycin**	Sigma-Aldrich	T7765; CAS No.: 11089-65-9
**OSMI-4**	MedChemExpress	HY-114361; CAS No.: 2260791-14-6
**5SGlcNHex**	Vocadlo Lab	Liu, TW, et al. *Angewandte Chemie* 2018
**Phalloidin Alexa Fluor 594**	Thermo	A12381; CAS No.: n/a
**GSK2606414**	MedChemExpress	HY-18072; CAS No.: 1337531-36-8
**Geldanamycin**	MedChemExpress	HY-15230; CAS No.: 30562-34-6
**GS-444217**	MedChemExpress	HY-100844; CAS No.: GS-444217
**Takinib**	MedChemExpress	HY-103490; CAS No.: 1111556-37-6
**URMC-099**	MedChemExpress	HY-12599; CAS No.: 1229582-33-5
**Ac4GalNAz**	Click Chemistry Tools	Cat# 1086; CAS No.: 653600-56-7
**Ac4GalNAlk**	Click Chemistry Tools	Cat# 1156; CAS No.: 1658458-26-4
**CalFluor 647 Azide**	Click Chemistry Tools	Cat# 1372; CAS No.: n/a
**THPTA (tris-hydroxypropyltriazolylmethylamine)**	Click Chemistry Tools	Cat#1010; CAS No.: 760952-88-3
**Biotin Azide Plus**	Click Chemistry Tools	Cat#1488; CAS No.: n/a
**Pierce High Capacity Streptavidin Agarose**	Thermo	Cat#20359; CAS No.: n/a

**Experimental models: Cell lines**		
**Human: HEK293 cell line**	ATCC	CRL-1573

**Experimental models: Organisms/strains**		
**Timed pregnant female rats, outbred (for NRVM isolation)**	Envigo	Hsd:Sprague Dawley Rat

Oligonucleotides		
**ds RNA sequence** targeting rat MKK3 (Map2K3):<br>top: rCrArUrGrGrArArGrCrUrArArUrArGrGrUrUrUrArCrUrUTG<br>bottom: rCrArArArGrUrArArArCrCrUrArUrUrArGrCrUrUrCrCrArUrGrArG	IDT DNA	Targeting Ex12 (3´UTR) of Map2k3<br>NM_001100674
**ds RNA sequence** targeting rat MKK6 (Map2K6):<br>top: rCrUrArCrArGrUrArGrUrUrGrArCrGrArGrArUrUrGrUrAAA<br>bottom: rUrUrUrArCrArArUrCrUrCrGrUrCrArArCrUrArCrUrGrUrArGrCrA	IDT DNA	Targeting Ex12 (3´UTR) of Map2k6<br>NM_053703
**ds RNA sequence** targeting rat Ask1 (Map3k5):<br>top: rArUrA rUrCrU rUrCrA rUrGrA rCrArA rUrCrA rGrArU rArGTC<br>bottom: rGrArC rUrArU rCrUrG rArUrU rGrUrC rArUrG rArArG rArUrA rUrUrU	IDT DNA	Targeting Ex17 of Map3k5<br>NM_001277694
**ds RNA sequence** targeting rat Tak1 (Map3k7):<br>top: rArArC rArGrU rUrArC rUrGrA rArUrU rGrUrU rUrUrA rUrGA T<br>bottom: rArUrC rArUrA rArArA rCrArA rUrUrC rArGrU rArArC rUrGrU rUrGrC	IDT DNA	Targeting Ex17 (3´UTR) of Map3k5<br>NM_001107920
**ds RNA sequence** targeting rat Mlk3 (Map3k11):<br>top: rArGrC rCrArA rUrGrA rArGrC rCrArA rArUrA rArArC rUrUG T<br>bottom: rArCrA rArGrU rUrUrA rUrUrU rGrGrC rUrUrC rArUrU rGrGrC rUrCrC	IDT DNA	Targeting Ex10 (3´UTR) of Map3k11<br>NM_001013150
**ds RNA sequence** targeting rat p47phox (Ncf1):<br>top: rArArG rCrArG rUrCrA rUrUrA rGrUrG rGrArU rArArU rGrCT T<br>bottom: rArArG rCrArU rUrArU rCrCrA rCrUrA rArUrG rArCrU rGrCrU rUrUrC	IDT DNA	Targeting Ex11 (3´UTR) of Ncf1<br>NM_053734
**ds RNA sequence** targeting rat gp91phox (Cybb):<br>top: rGrCrA rGrArA rGrGrU rGrGrU rCrArU rUrArC rCrArA rGrGT G<br>bottom: rCrArC rCrUrU rGrGrU rArArU rGrArC rCrArC rCrUrU rCrUrG rCrUrG	IDT DNA	Targeting Ex8/Ex9 of Cybb<br>NM_023965
**ds RNA sequence 1** targeting rat Tab1:<br>top: rArUrU rGrGrG rGrArU rUrArC rArArG rGrUrC rArArA rUrAT G<br>bottom: rCrArU rArUrU rUrGrA rCrCrU rUrGrU rArArU rCrCrC rCrArA rUrUrC	IDT DNA	Targeting Ex7 of Tab1<br>NM_001109976
**ds RNA sequence 2** targeting rat Tab1:<br>top: rArGrU rCrArA rCrArG rCrUrU rUrCrA rCrUrC rUrGrA rUrGA G<br>bottom: rCrUrC rArUrC rArGrA rGrUrG rArArA rGrCrU rGrUrU rGrArC rUrCrA	IDT DNA	Targeting Ex11 (3´UTR) of Tab1<br>NM_001109976

Software and algorithms		
**Fiji/ImageJ**	Schneider et al., 2012	https://imagej.nih.gov/ij/
**CellProfiler**	McQuin, Claire, et al. 2018	https://cellprofiler.org

Supporting Table S2. Primers used in real-time quantitative PCR

Target gene	Sequence (5´ to 3´)	Accession No.	Location
cTnT	GAG GAA GGC TGA AGA TGA GG	XM_039090351.1	Exon 11
	TCT CGC TCT GTC TGT CTC TT		Exon 13
Anp	ATC TGC CCT CTT GAA AAG CA	NM_012612.2	Exon 2
	AAG CTG TTG CAG CCT AGT CC		Exon 2
Bnp	GAT TCT GCT CCT GCT TTT CC	NM_031545.1	Exon 1
	CAT CGT GGA TTG TTC TGG AG		Exon 1
RCan1	ATT TTA GCT CCC TGA TTG CCT	NM_153724	Exon 1
	GAT GTC CTT GTC ATA CGT CCT		Exon 2
Gapdh	TCA AGA AGG TGG TGA AGC AG	NM_017008.4	Exon 7
	AGG TGG AAG AAT GGG AGT TG		Exon 7
36B4/Rplp0	TCC TGA GCG ATG TGC AGC TGA TAA	NM_022402.2	Exon 4/5
	GCC ATT GTC AAA CAC CTG CTG GAT		Exon 5
